# Supplementary material for: Improving lifestyle behaviours among women in Kisantu, the Democratic Republic of the Congo: A protocol of a cluster randomised controlled trial
Source: PLoS One. 2022 Sep 9;17(9):e0274517. doi: 10.1371/journal.pone.0274517 (PMC9462713; doi:10.1371/journal.pone.0274517)
Supplement: S2 File — (PDF) [file pone.0274517.s002.pdf]

ITM Clinical Trial

RESEARCH STUDY PROTOCOL

Prevention of type 2 diabetes and gestational diabetes among women of reproductive age in Kisantu, Democratic Republic of Congo

[KIS-ANTWERP]

Version 2.1
June 2021

Approved

Sponsor: Institute of Tropical Medicine
Nationalestraat 155
B-2000 Antwerp - Belgium

Coordinating Investigator: Prof. Dr. José L. Peñalvo

**Protocol Number:**	Not applicable
**ClinicalTrials.Gov.:**	Pending
**EudraCT Number**	Not applicable
**Study Title:**	Prevention of type 2 diabetes and gestational diabetes among women of reproductive age in Kisantu, Democratic Republic of Congo
**Study Acronym**	KIS-Antwerp
**Version:**	2.1
**Date:**	**21/06/2021**
**Previous version:**	Not applicable.
**Study drugs:**	Not applicable
**Funder:**	City of Antwerp
**Sponsor:**	Institute of Tropical Medicine, Antwerp, Belgium

Scientific supervision team

**Name**	**Institution**	**Function**	**Contact details**
José L. Peñalvo	ITM	Coordinating Investigator	jpenalvo@itg.be
Diana Sagastume	ITM	Clinical Researcher	dsagastume@itg.be
Deogratias Katsuva	ITM	Education coordinator and operational liaison	dkatsuva@itg.be

Operational team

**Name**	**Institution**	**Function**	**Contact details**
Felipe Sere	Memisa	Manager development funding at Headquarter	felipe.sere@memisa.be
Jean Clovis Kalobu	Memisa	Medical Advisor in DRC/ Principal Investigator	jean_clovis.kalobu@memisa-rdc.org
Olivier Kidima	Memisa	Projet Manager of Diabetes program in DRC	oli.kidima@memisa-rdc.org
Diertho Mputu	Memisa/ Partner (BDOM Kisantu)	Local project manager in Kongo Central	ed_kembo@yahoo.fr

Approved

Site study team

**Name**	**Institution**	**Function**	**Contact details**
Diertho Mputu	Memisa/BDOM Kisantu	Local project manager	ed_kembo@yahoo.fr
Jose Mavuna Nketo	District Manager Officer	Co-local project manager	josemavunanketo@gmail.com
EDDY Mbela	District Nurse supervisor	Data Manager	Eddymbela2020@gmail.com
Dimbelolo Jean Claude	Centre Education Diabète et Santé/PNLD	Head of Administration Board, and Deputy director of national program against Diabetes (PNLD)	dimbelolo@yahoo.fr
Sr. De Clerck	Centre Education Diabète et Santé	Responsible of CEDS	magguy.dc@gmail.com

Besides, local health professionals working at the healthcare centers of the project and also local peer educators will be involved in this study. Further characteristics regarding this figures are provided within this protocol.

Approved

STATEMENT OF COMPLIANCE

By signing this protocol, the Investigator(s) acknowledge(s) and agree(s):

This protocol contains the necessary information for conducting this clinical study. The Principal Investigator will conduct this study as detailed herein and will make every reasonable effort to complete the study within the time designated. The Principal Investigator commits to carry out the study in compliance with the protocol, amendments, applicable procedures and other study-related documents provided by the Sponsor, and in compliance with the Declaration of Helsinki, Good Clinical [Laboratory] Practice (GC[L]P), the EU General Data Protection Regulation 2016/679 (GDPR), the ESF/ALLEA Code of Conduct for Research Integrity, and applicable regulatory requirements.

The protocol and all relevant study information, which is provided by the Sponsor, will be made available to the physicians, nurses and other personnel who participate in conducting this study. The Investigator will use this material for their training so that they are fully informed regarding the drugs and the conduct of the study.

The Sponsor of this study – the Institute of Tropical Medicine in Antwerp, Belgium (ITM) – can at any time have access to the source documents from which Case Report Form information may have been generated and will be permitted to perform trial-related monitoring and audits. All study material will be maintained according to regulatory requirements and until the Sponsor advises that retention is no longer necessary.

Approved

COORDINATING INVESTIGATOR:

Title, Name: José L. Peñalvo Date:

Signed:

José Luis Peñalvo

Digitally signed by José Luis Peñalvo
Date: 2021.06.23 15:46:39 +02'00'

DIRECTOR OF THE INSTITUTE OF TROPICAL MEDICINE:

Title, Name: Dr. Marc-Alain Widdowson Date:

Signed:

Digitally signed by Marc-Alain Widdowson (Signature)
Date: 2021.07.06 13:31:16 +02'00'

PRINCIPAL INVESTIGATOR:

Title, Name: Jean Clovis Kalobu Kabundi Date: [illegible]

Signed: [illegible]

Signing this document, I commit to carry out the trial in accordance with the protocol, Good Clinical Practice and applicable ethical and regulatory requirements. I also acknowledge the paragraph relevant to study confidentiality and authorize the Institute of Tropical Medicine, Antwerp, Belgium to record my data on a computerized system containing all the data pertinent to the study.

TABLE OF CONTENTS

STATEMENT OF COMPLIANCE **4**
TABLE OF CONTENTS **6**
SYNOPSIS **7**
1.1 BACKGROUND 9
1.2 RATIONALE 10
2. STUDY OBJECTIVES **10**
2.1 PRIMARY OUTCOMES 11
2.2 SECONDARY OUTCOMES 11
3. STUDY DESIGN **12**
3.1 GENERAL STUDY DESIGN 12
3.2 BASELINE SURVEYS OR SUBSTUDIES (IF APPLICABLE) 14
4. PARTICIPANTS, POPULATION & SELECTION **14**
4.1 SETTINGS, SELECTION & RECRUITMENT 14
4.2 INCLUSION AND EXCLUSION CRITERIA 14
4.3 SAMPLE SIZE 14
4.4 RANDOMIZATION 15
4.5 WITHDRAWAL AND TERMINATION OF THE STUDY 16
5. STUDY PROCEDURES **17**
5.1 STUDY/VISIT SCHEDULE 17
5.2 OBTAINING INFORMED CONSENT 18
5.3 SPECIFIC PROCEDURES AND ACTIVITIES 19
5.4 LABORATORY PROCEDURES 23
6. STUDY INVESTIGATIONAL PRODUCT **23**
6.1 PURCHASING, PREPARATION AND ADMINISTRATION 23
6.2 PARTICIPANT COMPLIANCE MONITORING 24
6.3 PRIOR AND CONCOMITANT THERAPY 24
6.4 PACKAGING 24
6.5 RECEPTION, STORAGE, DISPENSING AND RETURN 24
7. SAFETY ASSESSMENT **24**
7.1 ADVERSE EVENTS 24
7.2 DATA AND SAFETY MONITORING BOARD 24
8. STATISTICAL METHODS **25**
8.1 STUDY HYPOTHESES AND HYPOTHESIS 25
8.2 VARIABLES OF INTEREST 25
8.3 STATISTICAL METHODS 25
8.4 SAMPLE SIZE AND POWER 26
9. MONITORING AND QUALITY ASSURANCE **28**
10. DATA MANAGEMENT **29**
11. ETHICAL ISSUES **30**
11.1 ETHICAL AND REGULATORY REVIEW 30
11.2 PROTOCOL AMENDMENTS 30
11.3 INFORMED CONSENT 30
11.4 CONFIDENTIALITY 30
11.5 RISKS AND BENEFITS 30
11.6 COMPENSATION FOR PARTICIPATION 31
11.7 INSURANCE 31
12. DISSEMINATION OF RESULTS, INTELLECTUAL PROPERTY **31**
13. ARCHIVING **32**
14. REFERENCES **33**
15. LIST OF ABBREVIATIONS **36**
16. ANNEXES **37**

Approved

SYNOPSIS

**HYPOTHESIS**	To evaluate whether the intervention group has a larger improvement in the healthy lifestyle questionnaire score than the comparison group, the following hypotheses were formulated:<br>- Null hypothesis (H_0): the score in the healthy lifestyle questionnaire is the same in the intervention and comparison group (or the intervention does not improve the score of the questionnaire).<br>- Alternative hypothesis (H_1): the score in the healthy lifestyle questionnaire is larger for the intervention group than the comparison group.
**DESIGN**	KIS-Antwerp will be implemented and evaluated using a matched cluster-randomized controlled trial involving 6 healthcare centers in Kisantu from where women will be recruited into two study groups: an intervention group and a comparison group.
**STUDY SITE & POPULATION**	The study will take place in 6 healthcare centers across Kisantu, Democratic Republic of Congo (DRC) and will be limited to women of reproductive age (18-49 years) irrespectively of pregnancy status. A total of 144 participants per arm necessary to detect a 12% change in the primary outcome with a level of significance of 0.05, power >80%, and considering a 20% loss of follow-up.
**DURATION**	The duration of the present study is 24 months.
**OBJECTIVES AND OUTCOMES**	This study aims to develop, implement, and evaluate a long-term program (KIS-Antwerp) focused on the prevention of type 2 diabetes mellitus (T2DM) and gestational diabetes (GDM) of women through healthy lifestyle promotion. The primary outcome is a healthy lifestyle score measured by closed-ended questionnaire. Secondary outcomes include anthropometric (weight, body mass index and waist circumference), clinical indicators (blood pressure and glycemia) and other dietary and physical activity questionnaires.
**INCLUSION & EXCLUSION CRITERIA**	To be eligible, study participants must meet the following criteria: Non-pregnant and pregnant (first trimester) women of reproductive age (18-49 years) who have not yet been diagnosed with type 1 diabetes or T2DM.<br>Exclusion criteria entail: Pregnant women with active GDM; Children and adolescents <18 years old; men; women of reproductive age with chronic conditions (*e.g.* chronic kidney disease); living outside the catchment area of the 6 healthcare centers; women without a permanent home or identified physical address; planning to move elsewhere in the following 2 years; women that do not provide a written informed consent
**SCREENING, RECRUITMENT & RANDOMIZATION**	Women of reproductive age (pregnant and non-pregnant) will be recruited primarily through the healthcare centers in Kisantu health district as they attend frequently to healthcare centers due to general consultations. The study is based on a hierarchical design where 2 groups of 3 healthcare centers balanced in population size coverage and geographical location are the units that are randomized to the intervention and comparison group through an algorithm allowing stratified allocation.

Approved

**STUDY INTERVENTION AND COMPARISON**	The intervention group will be provided with a preventive intervention program based on educational + motivational strategies including the following intervention program components: Individualized education, group education, physical activity sessions, setting goals, and usage of a pedometer. The comparison group will be limited to a basic educational strategy only (basic individual education). This design allows us to provide all women with proven effective preventive strategy (education) while testing for the added value of still experimental approaches (motivation).
**FOLLOW-UP**	Participants will be followed up for 24 months and measurements will be assessed every 6-months period (total of 5-time points: baseline + 4 follow up visits).
**ANALYTICAL METHODS**	Not applicable.
**SAFETY**	This study imposes a low risk for the participants as the intervention program compromises a lifestyle modification through education and motivational strategies and the measurements to be taken are non-invasive, with the exception of blood glucose assess by blood sampling using a finger prick. For this measurement, guidelines with all the necessary steps and equipment will be followed.
**STATISTICAL METHODS**	Demographic characteristics will be reported as measures of central tendency for continuous outcomes and frequency distribution and percentages for binary/categorical data. Consequently, demographic data will be explored for differences at baseline by comparing and testing means. After the termination of the study, the primary and secondary outcomes will be evaluated for normality by the Kolmogorov-Simonov test and visual inspection of histograms. For the primary ordinal outcome, the impact of the intervention program between baseline and follow up of the 2 groups (intervention and comparison group) will be assessed by using linear mixed models for repeated measurements. The same statistical model will be used to evaluate secondary outcomes.

Approved

1. Introduction

1.1 Background

Recent global figures from the World Health Organisation (WHO) indicate that the high prevalence of obesity is not just a problem of the industrialized countries, but also a raising concern in low and middle-income countries (LMIC), with more than 1.9 billion adults being overweight, of which 650 million obese[1]. This increase in the prevalence of obesity also indicates that many LMIC are now confronted with a double burden of disease were both still highly prevalent communicable and rising chronic non-communicable diseases (NCDs) co-exist[2,3]. In most countries in Sub-Sharan Africa (SSA), women are more likely to be obese or overweight than men[4,5]. Obese (Body mass index (BMI) >= 30 kg/m^2) women have a 28-times higher risk of developing T2DM than women with a normal weight[6]. Due to the increasing prevalence of obesity, it is expected that rates of T2DM will be also increasing among women in SSA[4,5]. Obesity is particularly alarming among women of reproductive age in urban SSA, among which rates have doubled or tripled over recent years[7]. The consequences of obesity on women of reproductive age particularly detrimental during pregnancy[7]. Obese women are recommended to gain only 11-20 pounds during pregnancy, in contrast to 25-25 in overweight and 25-35 pounds in women with normal weight[8]. The prevalence of maternal (pre-pregnancy or first trimester) obesity in SSA varies widely across countries from 6.5 to 50.7%, with older and multiparous women being more often affected[9]. Women whose weight gain during the first trimester of their pregnancy is outside the recommended ranges may be more at risk for GDM, a transitory condition but associated with serious health implications for both the mothers and the offspring[10]. Recent studies in SSA reported a pooled prevalence of T2DM in non-pregnant women of reproductive age of 7.2%[11]. Maternal overweight or obesity before conception is associated with the onset of GDM,[12] which prevalence is estimated to be 20.4% in Central Africa[13]. Importantly, GDM is associated with fetal macrosomia, perinatal mortality, preeclampsia, and caesarean delivery[14]. Studies have shown that women with a history of GDM have an increased risk of developing overt cardiovascular disease[15]. Furthermore, children born with macrosomia are more likely to develop obesity, T2DM, early cardiovascular disease, and certain cancers later in life[16–18]. With overlapping risk factors, T2DM and GDM can both be prevented by maintaining an optimal lifestyle, including adequate dietary habits and physical activity levels. A wealth of studies suggests that lifestyle modification strategies to prevent T2DM that are delivered under real-world conditions are effective for promoting weight loss[19,20], a known metabolic risk factor for T2DM[21,22]. There is also solid evidence on the effects of maintaining optimal diet and exercise habits during pregnancy in the reduction of the risk of GDM[23], highlighting the need for comprehensive (diet and physical activity) GDM preventive packages. Akin to the recommendations to increase physical activity for preventing T2DM, women at risk for GDM are recommended to do exercise at a moderate intensity, a minimum of three times a week for 30-60 min each time[24]. A recent study in the DRC found an overall prevalence of obesity, T2DM, and hypertension, of 4.5%, 11.7%, 18.2% respectively[25]. Women of reproductive age are a particularly vulnerable group, and there is a need for prevention programs that address the increasing incidence of prediabetes, T2DM, and GDM[11,13].

Approved

1.2 Rationale

The long-standing Kin-Antwerp project, sponsored by the City of Antwerp and supervised by the Institute of Tropical Medicine Antwerp (ITM) and operationalized by Memisa Belgium, has been successfully improving the lives of diabetic patients in Kinshasa. This established project encompasses 20 healthcare centers across the city, with an operational infrastructure equipped with experienced peer educators trained in diabetes management and infrastructure for an operational database for patient's follow up. During the last evaluation of the Kin-Anvers project in February 2020, overall positive feedback was gathered from participating partners, and several strengths were identified, among others: 1) The quality of the training curriculum of peer educators coordinated by the Centre d'Éducation Diabète et Santé (CEDS), 2) The decentralized diabetic-care structure, 3) The effectiveness of the appointment and monitoring system through the 'carnets de soins', and 4) The wholesome collaboration among stakeholders in diabetic care. Some areas of improvement were also observed, namely: 1) Partial usability of the operational database of T2DM patients to extract evidence to inform improved strategies, and 2) The dissimilar (or absent) approach of strategies for diabetes prevention across primary healthcare centers.

Based on the above, and in order to increase the preventive aspects of the program, and optimize the use of the limited available resources, the study partners opted for a refocus of the program. Due to the pivotal importance of placing preventive measures to decrease disease (diabetes) burden, particularly among vulnerable populations, and alleviate the health systems, we will build on the existing infrastructure of Kin-Antwerp to develop and implement an intervention program focused on the prevention of T2DM and GDM of women of reproductive age through lifestyle modification in Kisantu, DRC (KIS-Antwerp).

As in previous collaboration, ITM's role in this research is to provide scientific research guidance and supervision while Memisa is responsible for the operationalization and implementation of the study. Important to consider, the local health authorities (health district) will also cooperate in this project, as they are responsible for the primary health care at the operational level in DRC. These entities are in charge of developing health services and promote quality of care in all health facility. Due to these facts, the collaboration and involvement of the health district management team in Kisantu is an asset to our project, mainly for the monitoring of the sustainability of the project. Specific roles of the health district team entail intervention delivery, planning of service development as well as activities and resources, development of specific instructions or strategies, staff training, management, supervision and medical audit, operations research, quality assurance, review of hospital records and coordination.

2. Study Objectives

The overall objective of KIS-Antwerp is to develop, implement, and evaluate an intervention program for the prevention of T2DM and GDM among women in Kisantu (DRC) through the promotion of a healthy lifestyle and maintenance of healthy weight. After the evaluation, the intervention program will be made available for its integration in Kisantu's health systems, from where it could be potentially adapted to other areas in the country. To achieve this overall aim, the following specific objectives are planned:

1. Development of an effective strategy based on the promotion of healthy lifestyles to reduce the incidence/prevalence of overweight and obesity among women of reproductive age in Kisantu.

Approved

2. Development of an effective strategy based on the promotion of healthy lifestyles to increase the number of women who maintained a healthy weight gain during pregnancy corresponding to their pre-pregnancy weight.

To support these objectives and tackle potential weaknesses in the health system, structural and capacity-related objectives are also envisioned:

3. Improving the usability of data capturing systems for participant's follow-up and strategy-adaptation based on data analysis.

4. Improving knowledge among healthcare providers on effective strategies for the prevention of T2DM and GDM and their risk factors.

5. Improve the technique and precision of measurements concerning health visits among healthcare providers.

6. Improving women's attendance to antenatal care and therefore have an impact on maternal and neonatal health.

7. Promote a healthy lifestyle through the support of community-based strategies.

2.1 Primary Outcome

The primary outcome of this study is the adherence to a healthy lifestyle measured by a validated closed-ended questionnaire adapted to the context (**Annex 1**). This questionnaire emphasizes questions regarding an optimal diet and physical activity and it has been validated for weight control in lifestyle-related disease[26]. This questionnaire provides a final score ranging between 14-65 points. A lower score indicates poor adherence to a healthy lifestyle, while a high score indicates good adherence.

2.2 Secondary Outcomes

Other measurements include the anthropometry (weight, height, BMI, and waist circumference) as well as the clinical indicators of systolic blood pressure (SBP), diastolic blood pressure (DBP), and glycemic levels (this measurement will provide information on the absence or presence of pre-diabetes, T2DM, and GDM). Moreover, micronutrient adequacy will be evaluated by a dichotomous indicator of the minimum dietary diversity for women developed by the Food and Agriculture Organization of the United Nations (FAO)[27]. Also, physical activity frequency, duration, and type of physical activity will be assessed by using the International Physical Activity Questionnaire (IPAQ)-Short version[28].

To determine the possibility of complementing the intervention program with community based activities, close-ended Likert scale questionnaires with an agreement scale will be performed. Also, focus groups per healthcare center will be carried to understand the acceptability of community support and intervention perception.

Approved

3. Study Design

3.1 General study design

Intervention program

We will develop an educational-motivational-based intervention program to be provided at the healthcare center by community peer educators. The intervention program is based on the strategy for T2DM control implemented in Kinshasa (Kin-Antwerp), that will be tailored to the study participants characteristics and needs, and emphasizing the preventive component of T2DM and GDM through weight control and the promotion of a healthy lifestyle, optimal diet, and physical activity, as advised by the WHO [29]. As behavior change is challenging and motivational components have been associated with increased effectiveness in interventions that aim to promote diet and/or physical activity [30], motivational components will be integrated into the intervention program as well. Also, we aim to investigate during the first year of intervention if the intervention program can be complemented with community activities, as evidence has suggested that community participation is a fundamental element for positive public health impact [31]. If acceptable, community activities will be implemented in the second year of intervention program. The intervention program will be based on multicomponent interventions as it has been proven to be the most effective approach [32], mainly when targeting diet and/or physical activity [33]. The intervention program will include the components of: Individualized education, group education, physical activity sessions, goal setting, use of pedometer, and community activities if possible.

The delivery method of the intervention program and place to be provided are the following:

- Individualized method: healthcare centers and/or home visits
- Group method: healthcare centers
- Community method: parks, community room, home (second year of intervention program, if possible)

Evaluation study design

A cluster randomized controlled trial (cRCT) with a hierarchical design will be conducted whereby 2 groups of 3 healthcare centers (in total 6 healthcare centers) will be randomized to an intervention group or comparison group. The intervention group will be provided with the intervention program based on educational + motivational strategies whereas the comparison group will be limited to an basic educational strategy only (**Figure 1**). This design allows us to provide all women with proven effective preventive strategy (education) while testing for a comprehensive approach (education + motivation). The intervention program will last 24 months, starting approximately in September 2021 until September 2023 (Timeline of the study is presented in **Annex 2**).

The intervention program components and topics covered by the intervention group entail the following: Individualized education, group activities (group education and physical activity sessions), goal setting, use of pedometer, and community activities (if possible in the second year of intervention). In contrast, the components compromised in the comparison group are limited to basic individualized education at baseline only. Further information regarding the intervention program components and topics is presented in **Annex 3.**

Approved

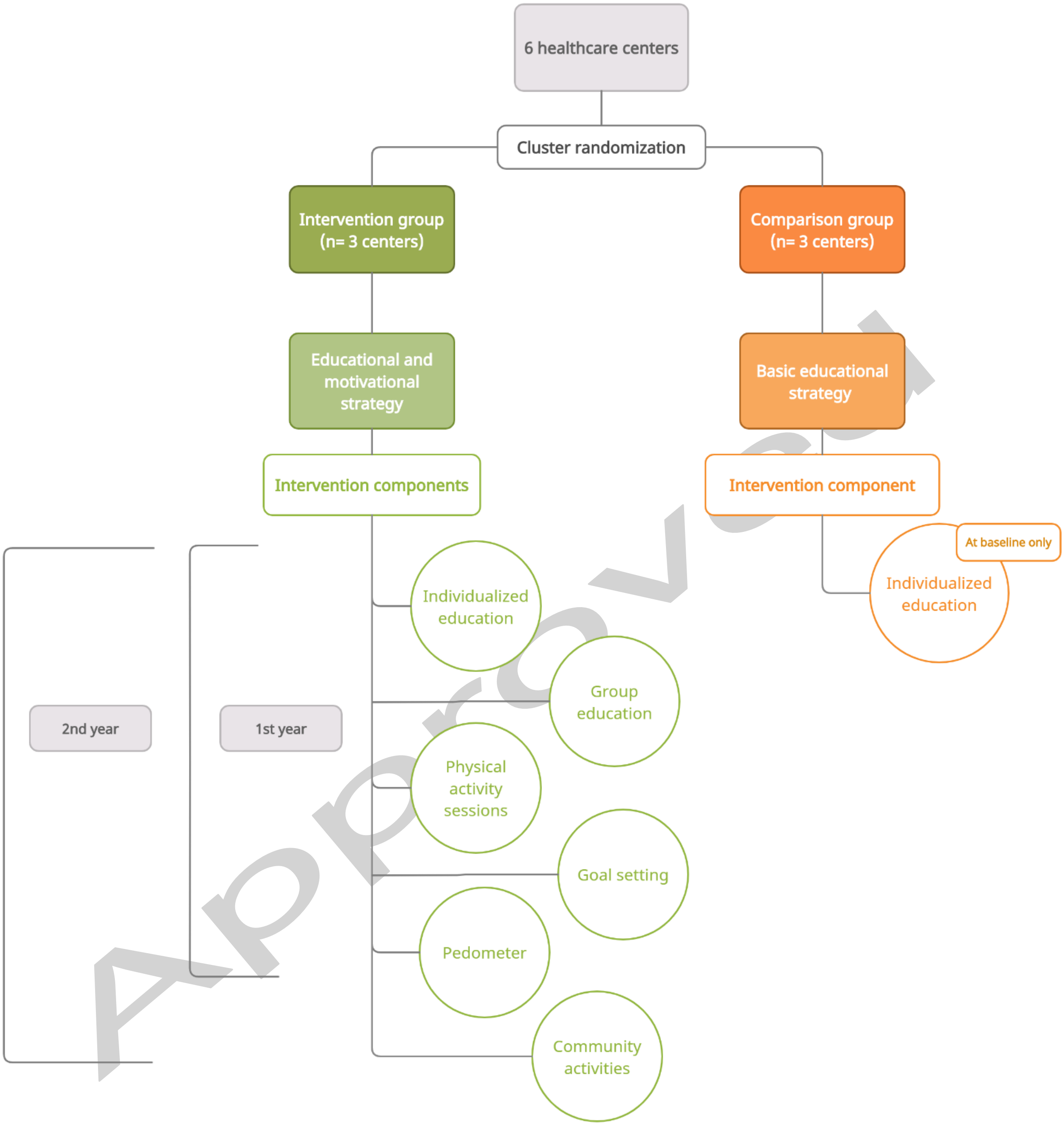

Figure 1. Study design and intervention program components

Approved

3.2 Baseline surveys or sub-studies (if applicable)

Not applicable.

4. Participants, Population & Selection

4.1 Settings, selection & recruitment

The study will be carried out in Kisantu health district (ZS Kisantu) encompassing a total population of 202,451 inhabitants. Six healthcare centers (NKANDU 1, KINTANU ETAT, NGEBA, KAVWAYA, KINKOKA, MADIMBA) covering approximately 107,000 inhabitants and sharing an integrated strategy of focused antenatal care (ANC) have been identified as operational/interventional centers. Based on past demographics [34], a total of 4,200 women per year are expected to be pregnant within the 6 centers population coverage, and of those approximately 92% will attend ANC at least in one occasion. Only a modest proportion of women (25%) are expected to attend the recommended 4 visits to ANC.

Women of reproductive age (pregnant and non-pregnant) will be recruited primarily through the healthcare centers in Kisantu as women attend frequently to healthcare centers due to general consultations for ANC and post-natal care, child monitoring, vaccinations among others. Within the 6 healthcare centers previously stated, health workers will identify women meeting the study legibility criteria and provide information about the study. If not enough women could be recruited via the healthcare centers consultations, informative posters will be displayed in the waiting rooms of the healthcare centers with a contact person for further information. If such posters are necessary and plan to be implemented, the posters will be submitted to the Ethical Committee in DRC and IRB at ITM for review and approval. Also community/village leaders will be approached and asked to encourage women to participate. Women interested in participating and meeting the eligibility criteria will be provided with detailed information about the intervention program, including all the specifics of the informed consent (IC). The recruitment of participants will be performed in 1 month, August 2021.

4.2 Inclusion and exclusion criteria

To be eligible, study participants must meet the following criterium:

- Non-pregnant or (first trimester) pregnant women of reproductive age (18-49 years old), without a previous diagnosis of type 1 diabetes or T2DM (confirmed by a random glucose test, which is a random blood test to check the glucose levels at random and no fasting condition is required. This test will be carried out by pricking the fingertip with a lancet and glucometer to all women during the recruitment).

Potential participants meeting any of the following criteria will not be enrolled in the study:

- Pregnant women with active GDM
- Adult males, and children and adolescents <18 years old of both sexes
- Pre-existent severe chronic conditions (e.g. chronic liver or kidney disease, chronic obstructive pulmonary disease (COPD))
- Living outside of the surrounding catchment areas of the 6 healthcare centers.
- Women internationally displaced, without a permanent home or identified physical address. This to decrease the possibility of drop-outs.
- Women planning to move elsewhere in the following 2 years
- Women that do not provide a written IC.

4.3 Sample size

The present study is based on a three-level hierarchical design where two groups of 3 healthcare centers (matched in population size and geographical area, urban/rural) will be randomized to the intervention and comparison group, and healthcare workers within the same cluster provide the intervention allocated to the participants
The sample size will be based on the expected differential change in the primary outcome of the study (healthy lifestyle score) determined by the questionnaire developed and validated in India by Dubasi and co-workers [26] to assess the adherence to dietary and physical activity advice for weight control in lifestyle-related diseases. The questionnaire's total score ranges between 14-65 points, a lower score indicates poor adherence to a healthy lifestyle, while a higher score indicates good adherence.

In this cRCT, 1 group of 3 healthcare centers will be randomly allocated to the intervention group and the other group of 3 healthcare centers to the comparison group. A comparable study measuring also lifestyle behavior changes as primary outcome, and using a similar score scale has reported a differential improvement from baseline to follow-up after 35 weeks of 20% between intervention and comparison groups [35]. Also, literature has reported intra-class correlation (ICC) coefficients for interventions concerning dietary and physical activity improvements range in between 0.007-0.3 [36,37].

Based on this previous evidence and the demographics of our study setting, the following assumptions have been used to approximate a power calculation estimate. This calculation was done using STATA version 16 for a cluster-randomized design assuming a realistic number of participants sample from each healthcare center whereby the following parameters were considered:

- 48 participants per cluster (144 participants per intervention program)
- Improvement of 12% in the lifestyle questionnaire score between intervention and comparison group (+ 6 score points)
- Effect size of 0.7 (score of the average person in the experimental group is 0.7 standard deviations above the average person in the comparison group, thus surpasses the scores of 69% of the comparison group).
- ICC of 0.1
- 20% loss to follow up and no attrition expected

Based on the above and considering a 95% level of significance (one-sided p-value), a power of 91% to detect the effect size is achieved. The possibility of an entire cluster withdrawal was also considered. Based on the previous parameters and 1 cluster dropped (2 intervention clusters and 3 comparison clusters or vice versa), a power of 85% would be reached.

4.4 Randomization

We will use matched randomization to ensure that the two groups are balanced in terms of population size coverage and geographical area (urban/rural). This will be performed by manually creating 2 groups, each of 3 healthcare centers, based on their population size coverage and geographical area followed by randomly assign the intervention and comparison group to the 2 balanced groups. The randomization will be performed before the intervention begins and it will be carried out by ITM's staff.

Due to the nature of this research and the impossibility of masking the intervention program, this study will not be blinded. Anyhow, to ameliorate the risk of bias, the cluster randomization design was chosen to avoid contamination of information between the participants per healthcare center.

Approved

4.5 Withdrawal and termination of the study

Reasons for Withdrawal

Participants may be withdrawn from the study for any reason at any time:

- The participant or legally acceptable representative withdraws the consent
- The Investigator judges that further participation would have a negative effect on the participant's health
- Participant changes location
- No longer interested
- Inability to continue follow-ups

Handling of Withdrawals

Participants can opt for withdrawal at any time for any reason. Participants will be provided with the following options:

1. Allow to be contacted by telephone for further follow-ups:
   - A: Continue with the collection of measurements by attending to the healthcare center
   - B: Provide an answer to self-reported measurements via telephone.
2. No longer be contacted about the research unless I need to be notified of a safety concern.

Also, participants will be provided on who to contact if there are any questions on concerns that arise after their participants of the study.

In the case participants develop T2DM or GDM, health professionals will ask the participant to return to the healthcare centers for monitoring the disease.

Definition of lost to follow-up/drop-outs.

When the health professional or peer educator has no news of the participants after 6 months (follow-up assessment), the health professional must take every effort to contact the participant by telephone to determine the reason of missing the follow-up assessment. If no news of the participant for 1 year and/or the participant missed 2 follow-up assessments, the health professional again must make every effort to contact the participant by telephone to establish the reason for the discontinuation of participation, and to suggest the participant comes to an end-of-study visit. If all these attempts to contact the participant fail, the investigator can then declare the participant "lost to follow-up". The investigator should document all these attempts in the corresponding study file.

Termination of Study

Reasons for termination of the study:

1. Impossible to meet the objectives.
2. Any unforeseen event (force majeure), as insecurity, displacement of the population, epidemic, strike.

If the study is prematurely terminated or suspended, the investigators will promptly inform the sponsors and the regulatory authority(ies) of the termination or suspension and the reason(s) for the termination or suspension. The EC's will also be informed promptly and provided the reason(s) for the termination or suspension by the Sponsor or by the investigator/institution, as specified by the applicable regulatory requirement(s).

Approved

5. Study Procedures

5.1 Study/visit schedule

The data collection of this study will be carried out by at least 2 health professionals recruited from each healthcare center (a minimum of 12 health professionals, female if feasible). Previous to the intervention program, health professionals will receive a guideline with standardized procedures for the measurements, to ensure the accuracy of the measurements and high-quality data. **Table 1** describes the schedule of measurements including the demographic characteristics and also the primary and secondary outcomes presented in section 2.1 and 2.2. It includes as well the tool to be used and the person that will assess the measurement.

Table 1. Schedule of measurements and tools

	Tool	*Administered by*	*Baseline*	*6 months*	*12 months*	*18 months*	*24 months*
**Demographics**							
Age	*Close ended questionnaire*	*Participant*	*X*				
Marital status			*X*				
Number of children			*X*				
Profession			*X*				
Smoking status			*X*				
**Healthy lifestyle adherence (Primary outcome)**							
Diet and physical activity questionnaire*	Closed-ended questionnaire	*Participant*	*X*	*X*	*X*	*X*	*X*
**Anthropometrics**							
Height**	*Height measuring board (SECA)*	*Health professional*	*X*				
Weight**[1]	*Digital weight scale (SECA)*		*X*	*X*	*X*	*X*	*X*
BMI**	*Calculation kg/m^2*		*X*	*X*	*X*	*X*	*X*
Waist circumference**	*Constant tension tape*		*X*	*X*	*X*	*X*	*X*
**Clinical indicators**							
Blood pressure**	*OMROM*[+]	*Health professional*	*X*	*X*	*X*	*X*	*X*
Glycemia**	*Achat glucometer (SECA)*		*X*	*X*	*X*	*X*	*X*
**Dietary assessment**							
Diet diversity**	*MDD-W questionnaire*	*Health professional*	*X*	*X*	*X*	*X*	*X*
**Physical activity**							
Physical activity questionnaire**	*IPAQ-short version*	*Health professional*	*X*	*X*	*X*	*X*	*X*
**Acceptability of community support (only intervention group)**							
Agreement scale questionnaire		*Health professional*		*X*	*X*		
**Intervention assessment (only intervention group)**							
Focus groups		*Peer educators and health professionals*		*X*	*X*	*X*	*X*

Abbreviations: Body mass index (BMI); Type 2 diabetes mellitus (T2DM); Gestational diabetes (GDM) ; Minimum dietary diversity for women (MDD-W)
In addition to these measurements, the ANC reports of pregnant women will be requested to the healthcare centers.
*Primary outcome. **Secondary outcome. + *OMRON:* Digital automatic blood pressure monitor. ~ 24-hour dietary recall will also be assessed randomly within the 6 month period, this will be performed by the health professional and by telephone. [1]1For pregnant women the weight will be compared to the recommended gain weight based on pre-pregnancy BMI, as follows: Underweight (BMI < 18.5): 13-18 kg; Normal weight (BMI 18.5 – 24.9): 11-16 kg; Overweight (25 - 29.9): 7-11 kg; Obese (BMI > 30): 5-9 kg.

Approved

5.2 Obtaining informed consent

As previously mentioned, health workers will identify women meeting the inclusion criteria and provide information about the study. Women interested in participating and meeting the inclusion criteria will be provided with further details about the intervention program. Consequently, the health workers will present an IC in French or in the local language (Kikongo or Lingala) and ask the potential participants to read it carefully and decide for participation by signing the ICF or refusal, contact involved staff for details if needed, or give it back within the following 2 weeks in case further consultation/time is needed.

To obtain IC of the participants, the IC form template (French version) developed by the ITM will be used stating the following (**Annex 4** for intervention group and **Annex 5** for comparison group):

Part 1. Research participant information form
This section will compromise a legitimate description of the purpose of the study, the procedures to be followed whereby detailed information of the activities and examinations that will take place will be provided. Also, it will explain the risks and benefits of participating in the study, as well as the compensation and insurance of the study. The form will also include information regarding privacy and data protection, also it will mention that the study was reviewed and approved by the Ethics Committee of the ITM and the local authorities of DRC. Moreover, study participants (or guardians) will be informed that participation in the study is completely voluntary and can withdraw from the study at any time without any negative consequences. Details regarding the contact person in case of questions will be also given.

This study informed consent is based on individuals capable of giving informed consent. For those unable of giving informed consent, we ensure that a legally authorized representative of this person has given permission and this permission takes account of the participants previously formed preferences and values. Minors (<18 years old) are not allowed to participate in this study. Moreover, this study has requested and obtained the permission of the gatekeeper, in this case Zones de Santé (health district) to enroll clusters (healthcare center) during the field visit happened 1st May – 9th May, 2021. Lastly, no opt-out procedures will be stated as this research does not entail any biological residuals nor other type of remaining material such as audio-recordings.

Part 2. Informed consent form
This part will entail the statement below and a space for the date, name, and signature of the participant. If a participant (or guardian) is unable to read or write, a signature from a witness to the informed consent discussion will be obtained.

> *"I have received a copy of the written participant information sheet. I also received verbally sufficient and understandable explanations, with enough time to ask questions, and my questions were satisfactorily answered.*
> *I freely consent to participate in this study and to cooperate in the study examinations/activities.*
> *I understand that during the study, my personal and medical data (or those of my child/the person I represent) will be recorded, and that the confidentiality of this data will be protected according to the national, European and Belgian applicable laws.*
> *I have also been informed that anonymized or pseudonymized data can be transferred to other countries than DRC".*

Approved

In this section, participants will be also asked for the permission of A) The use of their data for later research, given that it is in the context of this study, B) Informing other healthcare professionals about the participation in the study, C) They wish to be informed about any meaningful information important regarding their health, during or after the completion of the study.

5.3 Specific procedures and activities

In this section, the procedures for the measurements described in section 5.1 are presented. Additionally to the information found below, Standard operational procedures (SOPs) will be developed for each measurement adapted to the context.

Demographic characteristics

Baseline data will be collected at the beginning of the study by a 2-section questionnaire conducted by the health professional. Health professional will ask the questions and filled in the information provided by the participant. In section 1, the information to be gathered will include contact details (personal or relatives telephone number, email address, living address). This section of the questionnaire will always remain with the field staff, peer educators and healthcare professional, and will never be transferred into the data collection database nor shared with external bodies. Section 2 will include demographic characteristics of birthday, marital status, number of children, number of people living in the household, profession (also spouse), smoking (nonsmoker, former, or current), and frequency of alcohol consumption. All data collected by section 2 will be transferred to the database, pseudonymized directly and linked to the anonymized ID, to comply with EU GDPR and data protection regulations.

Adherence to a healthy lifestyle (primary outcome).

The healthy lifestyle adherence will be measured by a closed-ended multiple-choice questionnaire including questions regarding diet and physical activity. The questionnaire was developed in India by Dubasi and co-workers [26] to assess the adherence to dietary and physical activity advice for weight control in lifestyle-related diseases. It was validated with 100 patients with non-alcoholic fatty liver and concluded to be a reliable and valid tool to assess adherence to lifestyle modification advice in lifestyle-related diseases. This tool compromises 14 questions, 12 regarding diet and 2 related to physical activity. Each question has 5 multiple choice answers (A, B, C, D, E). The answers to each question were weighted based on relevance and interest of this study, providing a minimum response of 1 and a maximum of 5 points (**Annex 6**). The score of each question is summed up providing a final score ranging from 14 – 65 points.

The questionnaire will be conducted by the health professional. Health professional will ask the questions and filled in the answer provided by the participant. When finished, the questionnaire must be checked by the health professional for completeness. Consequently, the answer per question will be entered by the data-entry into an excel template developed by the ITM for the calculation of the final score after each follow-up.

Anthropometrics

The anthropometric measurements will be carried out by the health professional following the guideline for the collection of physical measurements advised by the WHO STEPwise approach to non-communicable disease risk factor surveillance [38]. These procedures entail the following:

Approved

Height

It will be measured by using a UNICEF height measuring board positioned on a firm surface against a wall. The procedure will be the following:

1. Ask the participant to
    - Remove their shoes and headgear (hat, cap, hairs bows, combs, etc.)
    - Stand on the board facing you
    - Stand with feet together, heels against the backboard, and knees straight
    - Look straight ahead and not look-up
2. Make sure eyes are at the same level as the ears
3. Move the measuring arm gently down on to the health of the participant and ask the participants to breathe in and stand tall
4. Read the height in centimeters at the exact point to the nearest mm
5. Ask the participant to step away from the measuring board
6. Record the height measurement in centimeters

Weight

It will be measured by a digital weight scale (SECA) placed on a firm, flat surface. The procedure will be the following:

1. Ask the participant to:
    - Remove footwear and socks. They should also take off heavy bets and empty their pockets of mobiles, wallets, and any other object.
    - Step on to scale with one foot on each side of the scale
    - Stand still, face forward, place arms on the side and wait until asked to step off
2. Record the weight in kg.

BMI

This measurement will be calculated by the health professionals by using the formula BMI= weight in kg/height in meters^2. Also, the BMI will be calculated automatically in the excel template.

Waist circumference

It will be evaluated with a constant tension tape (e.g. tape measure). Ideally, this measurement will be taken without clothing, directly over the skin. If not possible, the measurement may be taken over light clothing. Any thick or bulky clothing will be removed. The measurement should be assessed at the end of a normal expiration, with the arms relaxed at the sides, and at the midpoint between the lower margin of the last palpable rib and the top of the iliac crest (hip bone). The procedure will be the following:

1. Stand to the side of the participant, locate the last palpable rib and the top of the hip bone. You may ask the participant to assist you in locating these points on their body.
2. Ask the participant to wrap the tension tape around themselves and then position the tape at the midpoint of the last palpable rib and the top of the hip bone, making sure to wrap the tape over the same spot on the opposite side.
3. Ask the participant to:
    a. Stand with their feet together with weight evenly distributed across both feet
    b. Hold the arms in a relaxed position at the sides
    c. Breath normally for a few breaths, then make a normal expiration
4. Measure WC and read the measurement at the level of the tape to the nearest 0.1 cm, making sure to keep the measuring tape snug but not tight enough to cause compression of the skin

Approved

5. Record the measurement

<u>Clinical indicators</u>

These measurements will be taken by the health professionals following the guideline for the collection of physical measurements advised by the WHO STEPwise approach to non-communicable disease risk factor surveillance [38].

Glycemia

This biomarker will be measured as stated in the device instructions and with the equipment of device that measures glucose (Achat glucometer), a batch of sufficient reagent test strips, single-use lancets, gloves, cotton balls or swabs, and disposable container. The procedure will be the following:

1. Put on gloves
2. Remove a test strip, put it into the machine, and close the test strip box. The strips are sensitive to heat and humidity, so only take one strip at a time and close the box tightly
3. Rub and kneed a fingertip to help withdraw blood (rub the side of the participant's finger closest to the thumb)
4. Wipe or swab the fingertip by using a sterile swab.
5. Lance the massaged place on the fingertip with the lancing device
6. Allow a hanging blood drop to form without applying too much pressure
7. Carefully collect the blood with the capillary tube until the blood reaches the mark on the tube. Put the blood onto the test field without touching it
8. Give the participant a cotton ball to press on the puncture.
9. Wait for the measurement to be displayed. The results are usually displayed in mmol/L or mg/dL.
10. Record the result

Blood pressure

It will be assessed by an OMRON (Digital automatic blood pressure monitor)

The procedure will be the following:

Applying the cuff:

1. Place the left arm of the participant on the table with the palm facing upward
2. Remove or roll up clothing on the arm
3. Select the appropriate cuff size for the participant
4. Position the cuff above the elbow so that the lower band is positioned 1-2 cm above the elbow joint
5. Wrap the cuff snugly on to the arm and securely fasten with the Velcro
6. Keep the level of the cuff at the same level as the heart during the measurement

Using OMRON

1. Refer to the operating instructions included with the device.
2. Switch the monitor on
3. The monitor will start measuring when it detects the pulse. The SBP and DBP readings should be displayed within a few moments (systolic above and diastolic below)
4. Record the reading.
5. Wait three minutes, then repeat steps 2-5 two times more, take the mean of the second and third readings.

Approved

Dietary evaluation

Diet diversity

The minimum dietary diversity for women (MDD-W) developed by the Food and Agriculture Organization of the United Nations (FAO) is a dichotomous population-level indicator of whether participants have consumed at least five out of ten defined food groups the previous day [27]. The MDD-W is used as a proxy to describe one important dimension of women's diet quality (micronutrient adequacy) in national and subnational assessments.
The general ten groups are presented below (list of foods contained by each group is presented in section 2 of guideline [27]) :

1. Grains, white roots and tubers, and plantains
2. Pulses (beans, peas, and lentils)
3. Nuts and seeds
4. Dairy
5. Meat, poultry, and fish
6. Eggs
7. Dark green leafy vegetables
8. Other vitamin A-rich fruits and vegetables
9. Other vegetables
10. Other fruits

The threshold of this tool is: > 5 groups of food groups consumed indicates better micronutrient adequacy. The interpretation compromises that groups of women of reproductive age where a higher proportion consume food items from at least five of the ten food groups are likely to have higher micronutrient adequacy than other groups that have a lower proportion of women achieving the threshold of food items from at least five food groups. In other words, women of reproductive age who consume food items from five or more of the ten groups are also highly likely to consume at least one animal-source food and either pulses or nuts/seeds and food items from two or more of the fruit/vegetable food group. The MDD-W would be assessed using a questionnaire model (**Annex 7**) and following the guidelines by the FAO [27]. This questionnaire will be conducted and filled in by the health professional.

Physical activity assessment

This assessment will be performed with the use of the International Physical Activity Questionnaire – (IPAQ) Short Form [28]. This questionnaire compromises 7 questions and collects information on physical activity involvement on the domains of leisure time, domestic and gardening activities, and work-related and transport-related activities. Consequently, the Metabolic Equivalent (MET) per week will be calculated allowing to determine and classify the level of physical activity (low/medium/high) of the participant on a weekly basis. This questionnaire will be conducted and filled in by the health professional.

The questionnaire can be find at the following link https://journals.plos.org/plosone/article/file?type=supplementary&id=info:doi/10.1371/journal.pone.0219193.s010. The data will be analyzed according to the IPAQ guidelines presented in this link https://www.physio-pedia.com/images/c/c7/Quidelines_for_interpreting_the_IPAQ.pdf.

Approved

Acceptability of community-based strategies and intervention evaluation
To explore the feasibility of complementing the intervention program with community-based activities, close-ended Likert scale questionnaires with an agreement scale (Strongly disagree [1], disagree [2], undecided [3], agree [4], strongly agree [5]) will be performed twice during the first year of intervention. The questionnaire will be provided by the health professional to the participant from the 3 healthcare centers in the intervention group and it should be filled in by the participant. Also during our field visit, the identification of potential partners to be involved in the community activities will be carried out (e.g. community leaders and representatives, regulatory authorities, government agencies, etc.).

If feasible and acceptable, activities engaging the community will be integrated into the intervention program delivered to the intervention group. These activities will be guided by designated volunteer community leader/s, preferably enrolled participants from the intervention group. The designated community leader/s will be in charge of organizing and promoting healthy lifestyle activities with support from the peer educators and other bodies involved. If acceptable, potential activities to be integrated are: deliver material to promote healthy lifestyle, promote physical activity sessions (e.g. walking pathway), sharing healthy cooking recipes and experiences, healthy lifestyle education involving children and husbands, etc. As mentioned, the feasibility of community-based activities will be assessed during the first year of interventions, in which also the type of community activities will be define based on the needs and identified priorities by our target population.
As part of evaluating the feasibility of community-based activities but also to assess the intervention perception, focus groups with the participants per healthcare center of the intervention group will be carried out. The focus groups will provide the investigators' information regarding the perception of the intervention as well as identify needs (*e.g. including husbands or children*) and weaknesses that can be further addressed and improved. The focus groups will take place at each healthcare center of the intervention group every 6 months. All enrolled participants will be invited to participate in this activity by the peer educators and health professionals. Participants will be informed that this activity is completely voluntary, explained that the main objective is to discuss their perception regarding the intervention and to identify needs, weaknesses, and improvement points. Besides, participants will be informed about this activity before signing the ICF, where it will also emphasize that this activity is completely voluntarily, participation will have no impact in other related-activities to the intervention, and answers will remain anonymous. During the focus group, at each healthcare centers, all the attending participants will be divided into groups of a maximum of 10 people. Each group will have at least one moderator, a peer educator and/or healthcare professional, that will guide the questions, encourage discussion, and write down the most salient points of the discussion. The moderator will follow the focus group discussion (FGD) guide (**Annex 8**). After completion of each focus group, health professionals will synthetize this information and share it with the investigators for review.

5.4 Laboratory procedures

Not applicable.

6. Study Investigational Product

6.1 Purchasing, preparation and administration

Not applicable.

Approved

6.2 Participant compliance monitoring

Not applicable.

6.3 Prior and concomitant therapy

Not applicable.

6.4 Packaging

Not applicable.

6.5 Reception, storage, dispensing and return

Not applicable.

7. SAFETY ASSESSMENT

7.1 Adverse events

This is an intervention study with a low risk of adverse events as the intervention program compromises a lifestyle modification program through education and motivational strategies. The measurements to be taken are non-invasive, with the exception of blood glucose assess by blood sampling by using a finger prick. Moreover, the women participating in this study will be healthy (free of pre-existent severe conditions).

Concerning blood glucose measurement, its assessment will be performed by a health professional in a healthcare center and following the guideline developed by WHO STEPwise approach to non-communicable disease risk factor surveillance [38]. The measurement will also be taken by following the instruction of the device, with required equipment (see section 5.3). Moreover, as participants do not have T2DM either GDM, blood glucose will be assessed randomly at any time, meaning no fasting state is required.

Nonetheless, this study has a low risk of participants, health professionals will ask participants for any symptom, indicator, or manifestation has arisen throughout the period intervention/measurements. This will be recorded in the written formats where all the measurements are being collected approximately every 6 months by the health professionals and followed though resolution if necessary.

7.2 Data and Safety Monitoring Board

As this study presents a low-risk for the participants, a Data and Safety Monitoring board is not required. The safety evaluation of the intervention program and measurements will be performed by the health professionals during the assessment of measurements (~ 6 months) as stated in section 7.1.

Approved

8. Statistical Methods

The statistical analysis will be described in the statistical analysis plan written by the Coordinating Investigator and checked by a biostatistician, which is binding and will be finalized before database lock or before any other analysis takes place.

8.1 Study hypotheses and hypothesis

To evaluate whether the intervention group has a larger improvement in the healthy lifestyle questionnaire score than the comparison group, the following hypotheses were formulated:

- Null hypothesis (H_0): the score in the healthy lifestyle questionnaire is the same in the intervention and comparison group (or the intervention program does not improve the score of the questionnaire).
- Alternative hypothesis (H_1): the score in the healthy lifestyle questionnaire is larger for the intervention group than the comparison group.

8.2 Variables of interest

After the termination of the study, the primary and secondary outcomes will be evaluated for normality by the Kolmogorov-Simonov test and visual inspection of histograms. In case of non-normally distributed outcomes, log transformation and non-parametric tests will be considered.

The primary outcome (healthy lifestyle questionnaire score) will be a variable with ordinal values ranging between 14-65 points. The secondary outcomes of anthropometrics (weight [kg], height [m], BMI [kg/m^2], waist circumference [cm]), clinical indicators (glycemia [mg/dL] and blood pressure [mmHg]), and total MET-min/week and kcal/week collected by the physical activity questionnaire will be variables with continuous values. Only the outcome of diet diversity will be provided as an ordinal variables ranging from 0-10.

8.3 Statistical methods

8.3.1 Analysis populations

The analyses will be conducted based on the intention-to-treat principle. A probability of <0.05 will be considered statistically significant for all tests. All analyses will be conducted using STATA (Release 16/SE. College Station, TX: StataCorp LP) by ITM's staff.

8.3.2 Baseline characteristics

After the initiation of the study, demographic characteristics will be reported as measures of central tendency (mean and SD or median and interquartile range) for continuous outcomes and frequency distribution and percentages for binary/categorical data. Demographic data will be explored for differences at baseline by comparing and testing means of all demographic and baseline data.

8.3.3 Primary analysis

After the termination of the study, the primary and secondary outcomes will be evaluated for normality by the Kolmogorov-Simonov test and visual inspection of histograms. For the primary ordinal outcome (Healthy lifestyle Questionnaire Score), the impact of the intervention program

Approved

between baseline and follow up of the 2 groups (intervention and comparison group) will be assessed by using random effect (clusters) linear mixed models for repeated measurements.

8.3.4 Secondary and tertiary analysis

For secondary ordinal and continuous outcomes (anthropometric, clinical, questionnaires, etc.), exploratory analysis using the same method of linear mixed models for repeated measurements.

8.3.5 Subgroup analyses

To explore differences in the evolution between pregnant and non-pregnant women, subgroup analysis for the primary and secondary outcomes will be performed. Other potential subgroup analysis will compromise healthy and unhealthy subgroups based on BMI, glycemia and blood pressure at baseline to assess their evolution and identify the subgroups with larger and smaller effects on the primary and some secondary outcomes.

8.3.6 Multiplicity and Missing Data

Bonferroni's p-value correction for multiple comparisons will be considered. This will be performed by dividing the total p-value (0.05) by the final number of outcomes used in the final analyses (primary + secondary outcomes). Moreover, as mentioned the analysis will be performed under the ITT principle. To avoid and/or decrease missing data, particularly in the questionnaires, health professionals will be asked to revise the questionnaires for completeness right after the participants have filled them up. In the case of missing answers, participants will be asked to provide an answer to the questions. Nonetheless, the primary and secondary outcomes will be assessed by a linear mixed model, which is a model that can handle missing values by using maximum likelihood estimations.

Interim analyses will be performed for the primary and secondary parameters will be assessed after the completion of each set of measurements (~ 6 months) by quantifying the % of women improving dietary habits and physical activity levels and categorizing into different levels of improvement, % of women with a BMI <25 kg/m^2, % of pregnant women gaining the correct amount of weight based on their pre-pregnancy BMI, % of women with normal glycemia levels, and % of women with normal blood pressure. Also, evaluation of intervention program indicators will be performed though summarizing the % of trained peer educator and health professionals, % of optimal data available for hey indicators and the % of patients who have withdrawn the IC.

Moreover, to determine whether it is feasible and acceptable to complement the intervention program with community based-strategies, the Likert scale will be summarized by frequency distribution and percentages, this will be carried out during the first year of intervention program.

8.4 Sample size and power

Based on the evidence mentioned in section 4.3[36,37,39] and to explore different scenarios of the study regarding the primary outcome (healthy lifestyle questionnaire score), power calculation with a suitable range of assumptions regarding cluster sizes, score changes, intra-class correlations, and SDs were performed in STATA version 16 for a cluster-randomized design. All of the scenarios included a one-sided level of significance of 95%, cluster sizes of 20, 30, 40 participants per cluster, improvements in the score of 6%, 12% and 18%, intra-class correlations of 0.001, 0.01, 0.1, and 0.2, and SD of 5, 7, and 9 points. Power calculation with different scenarios are presented below.

Approved

Power calculation for **20 per cluster (60 participants per arm)**

	**Change of 3 points (6%)** (Effect size: 0.2-0.4)			**Change of 6 points (12%)** (Effect size: 0.4-0.9)			**Change of 9 points (18%)** (Effect size: 0.7-1.4)		
	SD 5	*SD 7*	*SD 9*	*SD 5*	*SD 7*	*SD 9*	*SD 5*	*SD 7*	*SD 9*
ICC: 0.001	0.95	0.75	0.56	1	1	0.98	1	1	0.99
ICC 0.01	0.91	0.69	0.51	1	0.99	0.96	1	1	0.99
ICC: 0.1	0.61	0.40	0.28	0.98	0.87	0.69	1	0.99	0.94
ICC:0.2	0.44	0.28	0.21	0.91	0.69	0.51	0.99	0.94	0.80

Power calculation for **30 per cluster (90 participants per arm)**

	**Change of 3 points (6%)** (Effect size: 0.2-0.4)			**Change of 6 points (12%)** (Effect size: 0.4-0.9)			**Change of 9 points (18%)** (Effect size: 0.7-1.4)		
	SD 5	*SD 7*	*SD 9*	*SD 5*	*SD 7*	*SD 9*	*SD 5*	*SD 7*	*SD 9*
ICC: 0.001	0.98	0.88	0.71	1	1	0.99	1	1	1
ICC 0.01	0.97	0.81	0.62	1	0.99	0.98	1	1	1
ICC: 0.1	0.67	0.43	0.30	0.99	0.89	0.73	1	0.99	0.96
ICC:0.2	0.45	0.29	0.22	0.93	0.71	0.53	0.99	0.95	0.82

Power calculation for **40 per cluster (120 participants per arm)**

	**Change of 3 points (6%)** (Effect size: 0.2-0.4)			**Change of 6 points (12%)** (Effect size: 0.4-0.9)			**Change of 9 points (18%)** (Effect size: 0.7-1.4)		
	SD 5	*SD 7*	*SD 9*	*SD 5*	*SD 7*	*SD 9*	*SD 5*	*SD 7*	*SD 9*
ICC: 0.001	0.99	0.95	0.81	1	1	1	1	1	1
ICC 0.01	0.98	0.88	0.70	1	1	0.99	1	1	1
ICC: 0.1	0.68	0.44	0.32	0.99	0.91	0.75	1	1	0.97
ICC:0.2	0.47	0.30	0.22	0.93	0.72	0.53	0.99	0.96	0.83

Approved

On average conditions, meaning 30 participants per cluster, change of 12%, ICC of 0.1 and SD of 7 points, a power of 89% would be reached. However, if one cluster withdrawal the power would decrease to 82%, borderline to detect a change with enough power. Hence, to ensure power considering a withdrawal of 1 cluster, sample size calculation presented in section 4.3 entails 40 participants per cluster plus 20% loss to follow, indicating 144 participants per arm (total of 288 participants enrolled in the study) are needed to detect a change of 12% (with ICC 0.1 and SD of 7) between the intervention and comparison group with a level of significance of 95%.

9. Monitoring And Quality Assurance

This study will be monitored in accordance with regulations applicable to clinical trials, including ICH-GCP and sponsor-specific SOPs for trainings, recruitment, intervention program, measurements and data management. The PI and involved site research staff will allocate adequate time and resources for such monitoring activities. The investigator will also ensure that the monitor is given access to all the above noted study-related documents and has adequate space and resources to conduct monitoring and source data verification.

Overall, 2 peer educators and 2 health professionals, per healthcare center meeting the characteristics presented in **Annex 9** will be recruited. Peer educators and health professionals will be provided with intervention program training where the topics and procedures for the intervention will be covered. In this regard, two different training will be provided. First, a 1-week training for health professionals only will be carried out in July 2021. This training will emphasize adherence to the protocol, measurement procedures and data collection. A second 1-week training will be provided to peer educators in August 2021 where they will be trained in the intervention program and delivery of the intervention. Both trainings will be given by the staff of Memisa and the health district, staff that has already been trained by the ITM during the first week of May 2021. Aside from the training, peer educators and health professionals will be provided with a manual containing all relevant information regarding the intervention program, objectives and description of roles for peer educators and health professionals, and the standard procedures for taking the measurements for the health professionals. Also, the data entry clerks will be trained on data entering and coding. Standard Operating Procedures (SOPs) will be developed for the trainings, recruitment, intervention program, measurements, and data management. The adherence to the SOPs will be evaluated by the coordinators of Memisa and randomly by the ITM staff.

Regarding data quality control, data will be assessed approximately every 6 months, after each set of measurements has been collected. The quantitative data quality control will be carried out by ITM staff using STATA/SE version 16. The following quality checks will be undertaken:

- Produced data: Data will be assessed for completeness, consistency, accuracy, and cleanness. Also the evolution of the primary and secondary parameters will be assessed based on % of women improving the lifestyle questionnaire score, % of women improving dietary habits and physical activity levels, % of women with a BMI <25 kg/m^2, % of pregnant women gaining the correct amount of weight based on their pre-pregnancy BMI, % of women with normal glycemia levels, and % of women with normal blood pressure.
- Intervention program indicators: this will be performed though summarizing the % of trained peer educator and health professionals, % of optimal data available for the primary and secondary outcomes, and the % of patients who have withdrawn from the study or loss to follow up.

Approved

Aside from quantitative quality controls, an intervention assessment based on focus groups of the participants will be carried out, as previously mentioned. These focus groups with the participants per healthcare center in the intervention group will take place every 6 months where the intervention perception, needs and weaknesses will be identified. Consequently, the information coming from this activity will be synthetize, reviewed and discussed by the investigators from Memisa and ITM, and take into account for further continuation of the intervention.

The local centers from Kisantu must allow the ITM's monitor or a monitor designated by the ITM, Dr. José L. Peñalvo and/or research assistant Diana Sagastume, access to any study-related files or source document present at the local center for external monitoring and control.

The sponsor will inform the Investigators concerned immediately upon notification of a pending study centers inspection by any regulatory authority or funder. Likewise, the investigator will inform the sponsor of any pending inspection.

10. Data Management

Data management procedures should comply with the ITM's Research Data Management and Data Sharing Policy.

Data will be collected in standardized written forms by the health professionals approximately every 6 months. After each set of measurements, the data will be entered into a locked Excel template (developed by the ITM staff) by the data entry clerk were personal identifiers will be deleted and replaced by an anonymous ID number (pseudonymization) and coding techniques will be used for traceable information (e.g. date of birth or name of healthcare center) assuring participants confidentiality. The name and any other identifying detail will be retained at the recruitment site only and will NOT be included in any study data electronic file. After the data from the participants has been entered per set of measurements (~6 months), the data will be securely managed and transferred from Kisantu to the ITM following procedures stated in the data sharing agreement. After the ITM has received the data, data quality control and analyses will be performed by transferring the data from the locked Excel file into the statistical package of STATA/SE version 16 (see section 9). This procedure will be repeated after the initiation of the study with the demographic data and repeated approximately every 6 months until the completion of the study. After completion of the study, the complete database will be locked. Consequently, the final analyses (see section 8) will be performed, followed by sharing the results with local entities of Kisantu, Ministry of Health of DRC, and with the Congolese Center of Knowledge Management in Kinshasa, as well as a scientific report/paper will be drafted.

Data will be processed in accordance with the European General Data Protection Regulation (GDPR) and with the Belgian legislation on the protection of natural persons concerning the processing of personal data, this in collaboration with the Data Proception Officer (DPO's) working on Secure File Transfer Protocol (SFTP) from the ITM.

The Investigator will retain all source study-related documents for each for 20 years after completion of the study (see section 13).

Approved

11. ETHICAL ISSUES

11.1 Ethical and regulatory review

This clinical trial will be submitted for formal review and approval to the Institutional Review Board (IRB) of the ITM and the Ethics Committee (EC) of DRC and, if needed Competent Authorities of DRC. No study-specific interventions will take place before written approval by the EC(s) has been obtained and the local regulatory requirements have been complied with, and the signature of the clinical study protocol of each contractual party involved has been obtained.

The study will be carried out according to the principles stated in the Declaration of Helsinki, all applicable regulations and according to the most recent GCP and GCLP guidelines.

The study will also be included in the Clinicaltrials.gov public registry prior to the start of participant recruitment.

11.2 Protocol amendments

Once the final clinical study protocol has been issued and signed by the authorized signatories, it cannot be informally altered. Protocol amendments have the same legal status and must pass through the appropriate steps before being implemented. Any substantial change must be approved by all the bodies and EC's that have approved the initial protocol, prior to being implemented, unless it is due to participant's safety concerns (in which case the immediate implementation can be necessary for the sake of participant's protection. In case modifications to the protocol or amendment are requested by any local EC during the review process, these must be discussed and agreed upon with the Sponsor prior to any resubmission incorporating those changes.

11.3 Informed consent

No participant may be enrolled into the study until the Investigator or designee has obtained the written IC form described in section 5.2.

11.4 Confidentiality

As mentioned, data will be processed in accordance with the GDPR and with the Belgian legislation on the protection of natural persons concerning the processing of personal data, this in collaboration with the Data Proception Officer (DPO's) working on Secure File Transfer Protocol (SFTP) from the ITM.

Privacy and confidentiality of the participants will be assured by deleting and replacing personal identifiers with an anonymous ID number and coding techniques for traceable information. Data will be stored in a secure electronic excel database and access will be granted only to researchers involved in this study and local entities of Kisantu. All study-related documents will be stored and retained for 20 years after completion of the study by the ITM staff. The primary findings of this study will be shared with local authorities of Kisantu and will be drafted for peer-reviewed scientific publication.

11.5 Risks and benefits

This study is a cRCT aiming to reduce the incidence/prevalence of T2DM and GDM in women of reproductive age attending 6 healthcare centers across Kisantu. This is an intervention study imposing

Approved

a low risk of participants as the intervention program compromises a lifestyle modification program through education and motivational strategies. Also the measurements to be taken are non-invasive, with the exception of blood glucose assess by blood sampling by using a finger prick. However, this measurement will be assessed following guidelines. Moreover, participants will benefit from acquiring knowledge concerning a healthy lifestyle for the prevention of the chronic condition. Besides participants will benefit from a closer follow up and individualized advice. In the case of participants get diagnosed with diabetes during the recruitment and ensuring to meet the inclusion criteria, the newly diagnosed patients will be provided with a printed digital booklet in French concerning information on diabetic control. This booklet was developed in previous collaborations (Kin-Antwerp).

As it is unethical to deprive relevant information for the well-being of women and also for the prevention of public health concerns as it is T2DM, this study entails an intervention group where a comprehensive educational and motivational strategy will be delivered compared to the comparison group where only a basic educational strategy will be provided. As this study includes women of reproductive age including non-pregnant, pregnant or breastfeeding women, women will be informed that this study will have no negative impact on their pregnancy, fetus, nor breastfeeding, controversially, this research will potential provide health benefits for the women and baby. Moreover, as this study is limited to women participants, the intervention program will be delivered by women peer educators and the measurements will be taken by women health professionals if possible, to respect cultural differences and gender sensitivity.

11.6 Compensation for participation

All measurements and intervention program material will be free of charge for the participants.
No reimbursement nor compensation will be offered to the participants. Women will be attending healthcare centers that are close to their living environment, thus easily accessible and reachable for the participants. No long distances nor long travels are expected, therefore, we expect no travel expenses from the participants.

11.7 Insurance

As required by the Belgian law on experiments on the human person of May 7th 2004, this study is covered by the umbrella insurance from ITM for low-risk studies as it is considered a class 2 study. This ITM Civil Liability Agreement for class 2 covers any harm, injury or (material) damage which may occur to study participants and which may be directly or indirectly caused by their participation in the trial.

The explicit mention of insurance should also be in the ICF with contact persons and contact details from the site staff. This should also be explained to the participants during the IC verbal procedure, but it should be clear to the participants that it only covers any study-related injury or inconvenience in order to prevent abuse.

12. DISSEMINATION OF RESULTS, INTELLECTUAL PROPERTY

A digital report including the output of this study will be shared with local entities from Kisantu, Ministry of Health of DRC, and with the Congolese Center of Knowledge Management in Kinshasa, as they provide benefits for the local population. Moreover, the results will offer advice for potential health policy implementation for the prevention or preventive strategies for NCDs. Additionally, the

Approved

findings will be drafted into a scientific manuscript in accordance with the CONSORT statement in collaboration between the ITM, Memisa, and local entities of Kisantu, and submitted for peer-review.

13. ARCHIVING

The sponsor and Investigator must maintain adequate and accurate records to enable the conduct of the study to be fully documented and the study data to be verified. The relevant (essential) documents are those documents which individually and collectively permit to assess the conduct of the trial, the quality of the data produced and the compliance with GCP standards and applicable regulatory requirements. The Investigator's File should at least contain all the (essential) documents as listed in the procedure "Set up and maintenance of the Investigator Trial File". A copy of all source data and Case Report Forms must always be kept on site. Only ITM, Memisa and local entities of Kisantu staff that are competent and delegated to work for the study will be got access to the study files.

All the relevant study documentation present at all partners involved should be retained for a minimum of twenty years and according to applicable local regulations. The Sponsor should be informed prior to the destruction of the files. After completion of the study, the Investigators File will remain available for internal audits and/or inspections of regulatory authorities for a period of twenty years, unless differently requested by national authorities.

Approved

14. REFERENCES

1 WHO. Obesity and overweight. https://www.who.int/news-room/fact-sheets/detail/obesity-and-overweight (accessed 21 Oct 2020).
2 Boutayeb A, Boutayeb S. The burden of non communicable diseases in developing countries. *Int J Equity Health* 2005;4:2. doi:10.1186/1475-9276-4-2
3 WHO. Technical Report Series: Part III: Report of a WHO Consultation; 2000. Obesity: Preventing and managing the global Epidemic. 2000.
4 Imoisili OE, Sumner AE. Preventing diabetes and atherosclerosis in sub-Saharan Africa: Should the metabolic syndrome have a role? *Curr Cardiovasc Risk Rep* 2009;3:161–7. doi:10.1007/s12170-009-0026-7
5 BeLue R, Okoror TA, Iwelunmor J, *et al.* An overview of cardiovascular risk factor burden in sub-Saharan African countries: A socio-cultural perspective. *Global Health* 2009;5:10. doi:10.1186/1744-8603-5-10
6 Barnes AS. The epidemic of obesity and diabetes: Trends and treatments. *Texas Hear Inst J* 2011;38:142–4.www.cdc.gov/diabetes/pubs/pdf/ndfs_2007.pdf. (accessed 21 Oct 2020).
7 Amugsi DA, Dimbuene ZT, Mberu B, *et al.* Prevalence and time trends in overweight and obesity among urban women: An analysis of demographic and health surveys data from 24 African countries, 1991 - 2014. *BMJ Open* 2017;7:e017344. doi:10.1136/bmjopen-2017-017344
8 US Center for Disease Control. Weight Gain During Pregnancy. 2019.https://www.cdc.gov/reproductivehealth/maternalinfanthealth/pregnancy-weight-gain.htm (accessed 21 Oct 2020).
9 Onubi OJ, Marais D, Aucott L, *et al.* Maternal obesity in Africa: A systematic review and meta-analysis. *J Public Heal (United Kingdom)* 2016;38:e218–31. doi:10.1093/pubmed/fdv138
10 Hedderson MM, Gunderson EP, Ferrara A. Gestational weight gain and risk of gestational diabetes mellitus. *Obstet Gynecol* 2010;115:597–604. doi:10.1097/AOG.0b013e3181cfce4f
11 Chivese T, Werfalli MM, Magodoro I, *et al.* Prevalence of type 2 diabetes mellitus in women of childbearing age in Africa during 2000-2016: A systematic review and meta-analysis. *BMJ Open* 2019;9:e024345. doi:10.1136/bmjopen-2018-024345
12 Okosun IS, Chandra KMD, Boev A, *et al.* Abdominal adiposity in U.S. adults: Prevalence and trends, 1960-2000. *Prev Med (Baltim)* 2004;39:197–206. doi:10.1016/j.ypmed.2004.01.023
13 Muche AA, Olayemi OO, Gete YK. Prevalence and determinants of gestational diabetes mellitus in Africa based on the updated international diagnostic criteria: A systematic review and meta-analysis. *Arch Public Heal* 2019;77:36. doi:10.1186/s13690-019-0362-0
14 Wendland EM, Torloni MR, Falavigna M, *et al.* Gestational diabetes and pregnancy outcomes - a systematic review of the World Health Organization (WHO) and the International Association of Diabetes in Pregnancy Study Groups (IADPSG) diagnostic criteria. *BMC Pregnancy Childbirth* 2012;12:23. doi:10.1186/1471-2393-12-23
15 Carr DB, Utzschneider KM, Hull RL, *et al.* Gestational diabetes mellitus increases the risk of cardiovascular disease in women with a family history of type 2 diabetes. *Diabetes Care* 2006;29:2078–83. doi:10.2337/dc05-2482
16 Boney CM, Verma A, Tucker R, *et al.* Metabolic syndrome in childhood: Association with birth weight, maternal obesity, and gestational diabetes mellitus. *Pediatrics* 2005;115:2020. doi:10.1542/peds.2004-1808
17 Ahlgren M, Wohlfahrt J, Olsen LW, *et al.* Birth weight and risk of cancer. *Cancer* 2007;110:412–9. doi:10.1002/cncr.22773
18 Samaras TT, Elrick H, Storms LH, *et al.* Birthweight, Rapid growth, Cancer, and Longevity: A

Approved

Review. *J Natl Med Assoc* 2003;95.

19 Cardona-Morrell M, Rychetnik L, Morrell SL, *et al.* Reduction of diabetes risk in routine clinical practice: Are physical activity and nutrition interventions feasible and are the outcomes from reference trials replicable? A systematic review and meta-analysis. *BMC Public Health* 2010;10:653. doi:10.1186/1471-2458-10-653

20 Ali MK, Echouffo-Tcheugui J, Williamson DF. How effective were lifestyle interventions in real-world settings that were modeled on the diabetes prevention program? *Health Aff* 2012;31:67–75. doi:10.1377/hlthaff.2011.1009

21 Abdullah A, Peeters A, de Courten M, *et al.* The magnitude of association between overweight and obesity and the risk of diabetes: A meta-analysis of prospective cohort studies. *Diabetes Res Clin Pract* 2010;89:309–19. doi:10.1016/j.diabres.2010.04.012

22 Cloostermans L, Wendel-Vos W, Doornbos G, *et al.* Independent and combined effects of physical activity and body mass index on the development of Type 2 Diabetes - a meta-analysis of 9 prospective cohort studies. *Int J Behav Nutr Phys Act* 2015;12:147. doi:10.1186/s12966-015-0304-3

23 Guo XY, Shu J, Fu XH, *et al.* Improving the effectiveness of lifestyle interventions for gestational diabetes prevention: a meta-analysis and meta-regression. BJOG An Int. J. Obstet. Gynaecol. 2019;126:311–20. doi:10.1111/1471-0528.15467

24 Padayachee C. Exercise guidelines for gestational diabetes mellitus. *World J Diabetes* 2015;6:1033. doi:10.4239/wjd.v6.i8.1033

25 Mawaw PM, Yav T, Mukuku O, *et al.* Prevalence of obesity, diabetes mellitus, hypertension and associated risk factors in a mining workforce, Democratic Republic of Congo. *Pan Afr Med J* 2017;28. doi:10.11604/pamj.2017.28.282.14361

26 Dubasi S, Ranjan P, Arora C, *et al.* Questionnaire to assess adherence to diet and exercise advices for weight management in lifestyle-related diseases. *J Fam Med Prim Care* 2019;8:689. doi:10.4103/jfmpc.jfmpc_338_18

27 FAO and FHI 360. Minimum Dietary Diversity for Women- A Guide to Measurement. Rome: 2016. www.fao.org/publications (accessed 22 Oct 2020).

28 Craig CL, Marshall AL, Sjöström M, *et al.* International physical activity questionnaire: 12-Country reliability and validity. *Med Sci Sports Exerc* 2003;35:1381–95. doi:10.1249/01.MSS.0000078924.61453.FB

29 WHO. Diabetes. 2020.https://www.who.int/news-room/fact-sheets/detail/diabetes (accessed 21 Oct 2020).

30 Greaves CJ, Sheppard KE, Abraham C, *et al.* Systematic review of reviews of intervention components associated with increased effectiveness in dietary and physical activity interventions. *BMC Public Health* 2011;11:119. doi:10.1186/1471-2458-11-119

31 Haldane V, Chuah FLH, Srivastava A, *et al.* Community participation in health services development, implementation, and evaluation: A systematic review of empowerment, health, community, and process outcomes. *PLoS One* 2019;14. doi:10.1371/journal.pone.0216112

32 WHO. Interventions on diet and physical activity. What works? Summary Report. In: WHO, ed. Geneva: 2009.

33 Schröer S, Haupt J, Pieper C. Evidence-based lifestyle interventions in the workplace-an overview. *Occup Med (Chic Ill)* 2014;64:8–12. doi:10.1093/occmed/kqt136

34 Mavuna J. Presentation de la Zone de Sante de Kisantu - Revue Annuel 2019. 2020.

35 Phelan S, Wing RR, Brannen A, *et al.* Randomized controlled clinical trial of behavioral lifestyle intervention with partial meal replacement to reduce excessive gestational weight gain. *Am J Clin Nutr* 2018;107:183–94. doi:10.1093/ajcn/nqx043

36 Thompson DM, Fernald DH, Mold JW. Intraclass correlation coefficients typical of cluster-randomized studies: Estimates from the robert wood johnson prescription for health projects.

Approved

Ann Fam Med 2012;10:235–40. doi:10.1370/afm.1347

37 Masood M, Reidpath DD. Intraclass correlation and design effect in BMI, physical activity and diet: A cross-sectional study of 56 countries. *BMJ Open* 2016;6. doi:10.1136/bmjopen-2015-008173

38 WHO. WHO STEPSwise approach to noncommunicable disease risk factor surveillance. 2017.

39 Pinto AM, Fava JL, Raynor HA, *et al.* Development and validation of the weight control strategies scale. *Obesity* 2013;21:2429–36. doi:10.1002/oby.20368

40 Global Nutrition Report. Democratic Republic of the Congo Nutrition Profile. 2019. doi:10.1038/s41586-019-1878-8

41 WHO. Healthy diet. 2020.https://www.who.int/news-room/fact-sheets/detail/healthy-diet (accessed 21 Oct 2020).

42 Metro South Health. Congolese food and cultural profile: dietetic consultation guide 1. Booking a client appointment 2. Preparation for the consultation Working with an interpreter. 2015.

43 Harvard T.H. Chan School of Public Health. Healthy Eating Plate | The Nutrition Source . 2011.https://www.hsph.harvard.edu/nutritionsource/healthy-eating-plate/ (accessed 21 Oct 2020).

44 WHO. Physical activity. 2018.https://www.who.int/news-room/fact-sheets/detail/physical-activity (accessed 23 Oct 2020).

45 WHO. Physical activity. 2018.https://www.who.int/news-room/fact-sheets/detail/physical-activity (accessed 21 Oct 2020).

Approved

15. LIST OF ABBREVIATIONS

ANC	Antenatal Care
BMI	Body Mass Index
cRCT	Cluster-Randomized Controlled Trial
DBP	Diastolic Blood Pressure
DPO	Data Protection Officer
DRC	Democratic Republic of Congo
EC	Ethics Committee
FAO	Food and Agriculture Organization of the United Nations
GDM	Gestational Diabetes Mellitus
GDPR	General Data Protection Regulation
IC(F)	Informed Consent (Form)
ICC	Intra-Class Correlation
IRB	Institutional Review Board
ITM	Institute of Tropical Medicine of Antwerp
MDD-W	Minimum Dietary Diversity for Women
PI	Principal Investigator
SBP	Systolic Blood Pressure
SFTP	Secure File Transfer Protocol
SOPs	Standard Operating Procedures
SSA	Sub-Saharan Africa
T2DM	Type 2 Diabetes Mellitus
WHO	World Health Organization

Approved

16. ANNEXES

Annex 1. *Healthy lifestyle questionnaire (Primary outcome)*

QUESTIONNAIRE D'ENQUETE ETUDE DIABETE KISANTU

Q1) Combien de fois mangez-vous par jour (y compris thé, café, fruits, légumes, manioc, patate douce, tarot, igname, arachides grains grillés ou sous forme de pate pain complet)?

A) >6 fois
B) 6 fois
C) 5 fois
D) 4 fois
E) 3 fois

Q2) A quelle fréquence buvez-vous des boissons sucrées comme les boissons gazeuses, le jus etc.?

A) Au moins 1 fois par jour
B) 3 à 6 fois par semaine
C) 1 à 2 fois par semaine
D) 2 à 3 fois par mois
E) 1 fois le mois ou moins

Q3) A quelle fréquence mangez-vous des aliments sucrés comme le gâteau, cake, confiture, chocolat, bonbons, biscuits etc.?

A) Au moins 1 fois par jour
B) 3 à 6 fois par semaine
C) 1 à 2 fois par semaine
D) 2 à 3 fois par mois
E) 1 fois le mois ou moins

Q4) A quelle fréquence mangez-vous des aliments frits comme les beignets, patate douce, banane plantin, frites à base de manioc, dinde (croupion), pomme terre frites, galette?

A) Au moins 1 fois par jour
B) 3 à 6 fois par semaine
C) 1 à 2 fois par semaine
D) 2 à 3 fois par mois
E) 1 fois le mois ou moins

Q5) A quelle fréquence mangez-vous des aliments salés comme popcorn, poisson salé etc.?

A) Au moins 1 fois par jour
B) 3 à 6 fois par semaine
C) 1 à 2 fois par semaine
D) 2 à 3 fois par mois
E) 1 fois le mois ou moins

Q8) À quelle fréquence mangez-vous des légumineuses comme le haricot, petit pois, courge, sésame et les légumes vert comme feuille de manioc, épinards, fumbwa, oseilles ?

A) A tout moment pendant les grands repas
B) Au moins 1 fois par jour
C) 3 à 4 fois par semaine
D) 1 fois par semaine
E) Moins d'1 fois par semaine

Q9) À quelle fréquence mangez-vous des graisses saturées comme la graisse de mouton, les jaunes d'œufs, le beurre, la margarine, etc.?

A) Au moins 1 fois par jour
B) 3 à 6 fois par semaine
C) 1 à 2 fois par semaine
D) 2 à 3 fois par mois
E) 1 fois le mois ou moins

À quelle fréquence mangez-vous des aliments riches en graisse combiner aux aliments comme châteaux avec mayonnaise, charcuterie, omelette+ une boisson sucrée (l'une de chose peut manquer mais principalement les châteaux et boisson sucré fréquent ?

A) Au moins 1 fois par jour
B) 3 à 6 fois par semaine
C) 1 à 2 fois par semaine
D) 2 à 3 fois par mois
E) 1 fois le mois ou moins

Q11) À quelle fréquence mangez-vous du beurre, de la crème, de la mayonnaise, etc.?

A) Au moins 1 fois par jour
B) 3 à 6 fois par semaine
C) 1 à 2 fois par semaine
D) 2 à 3 fois par mois
E) 1 fois le mois ou moins

Q12) À quelle fréquence mangez-vous à l'extérieur de la maison (comme un mariage, une fête, une activité familiale, deuil etc.)?

A) Plus de 3 fois par semaine
B) Plus d'une fois par semaine
C) 2 fois par mois
D) 1 fois par mois
E) Moins d'1 fois par mois

Approved

Q6) Combien de fois consommez-vous du sucre ou du miel dans votre thé ou café etc.?

A) Au moins 1 fois par jour
B) 3 à 6 fois par semaine
C) 1 à 2 fois par semaine
D) 2 à 3 fois par mois
E) 1 fois le mois ou moins

Q7) À quelle fréquence mangez-vous des fruits et légumes?

A) A tout moment pendant les grands repas
B) Au moins 1 fois par jour
C) 3 à 4 fois par semaine
D) 1 fois par semaine
E) Moins d'1 fois par semaine

Q13) Combien de jours d'exercice physique faites-vous par semaine?

A) Chaque jour
B) 5 à 6 fois par semaine
C) 3 à 4 fois par semaine
D) 1 à 2 fois par semaine
E) Jamais

Q14) Combien de temps d'exercice faites-vous pour chaque session?

A) >40 minutes
B) 30–40 minutes
C) 20–30 minutes
D) 20–10 minutes
E) <10 minutes

Annex 2. *Timeline of the study*

		**2020**			**2021**			**2022**			**2023**		
**Phase**		T1	T2	T3	T1	T2	T3	T1	T2	T3	T1	T2	T3
**Ethical Clearance**													
**Preparation of tools**													
**Training**													
**Recruitment**													
**Intervention**													
**Evaluation**													
**Data analysis**													
**Dissemination of results**													

Approved

Annex 3. *Intervention components and topics covered by T2DM and GD preventive intervention program*

Intervention program components	Topics
Individualized education (both groups)	
Dietary advice based on dietary needs of DRC [40]	- Food groups and food sources<br>- Food choices: WHO healthy diet guidelines [41] considering the Congolese food and cultural profile: dietetic consultation guideline [42]<br>- Plate distribution; adapted to foods frequently consumed in Kisantu [43]<br>- A healthy diet during pregnancy
Physical activity	- WHO physical activity advice and examples [44]<br>- Physical activity during pregnancy [24]
Educational material	- Topics above.
Group education (intervention group)	
T2DM and GDM [29]	- Definition of T2DM and GDM<br>- Complications<br>- Prevention
Benefits of physical activity [45]	- Weight control<br>- Prevention of NCDs
Cooking oils and preparation techniques	- Cooking methods<br>- Cooking oils<br>- Benefits and risks<br>- Example of healthy cooking methods
Alcohol and tobacco use	- Risks of excessive use
Healthy weight gain during pregnancy	- Benefits and risks<br>- How to reach a healthy weight gain
Educational material	- Topics above.
Physical activity classes (intervention group)	
Work-out sessions	- Walking pathway<br>- Aerobics class<br>- Dancing class
Setting goals (intervention group)	
Personal goals	- Setting individualized dietary goals<br>- Setting weight goals<br>- Setting physical activity goals
Pedometer (intervention group)	
	- Individual explanation (incorporated to the individual component): How to use a pedometer<br>- Use a pedometer
Community based activities (intervention group)	
Community activities	- Sharing cooking recipes<br>- Physical activity classes<br>- Educational class with family<br>- Sharing personal journey

Approved

Annex 4. *Informed consent form for participants in the intervention group*

Partie 1. FORMULAIRE D'INFORMATION PARTICIPANTE À L'ÉTUDE

Prévention des diabètes type 2 et gestationnel chez les femmes en âge de procréer à Kisantu, RD Congo

Vous êtes invitée à participer à une étude sur la prévention des diabètes type 2 et gestationnel chez la femme en âge de procréer, réalisée par MEMISA en collaboration avec l'Institut de Médecine Tropicale d'Anvers. Avant de décider de participer ou non à cette étude, il est important que vous compreniez les informations contenues dans ce formulaire. Ce formulaire explique vos droits, nos responsabilités, le but, les procédures, les avantages, risques et inconvénients éventuels, et votre droit de retirer votre consentement à tout moment.

N'hésitez pas à poser des questions, par exemple sur les avantages et risques éventuels concernant cette étude. Votre participation est entièrement volontaire. Vous pouvez parler de l'étude avec n'importe qui et prendre le temps de réfléchir à votre participation. Même si vous renoncez à participer à cette étude, vous continuerez à bénéficier des soins de qualité au sein de la formation sanitaire. Cependant, si vous choisissez d'y participer, vous devez toujours garder sur vous ces informations générales et le formulaire signé pendant toute la durée de l'étude.

OBJET ET DESCRIPTION DE L'ÉTUDE

Cette étude est menée pour en savoir plus sur la prévention du diabète chez la femme enceinte ou en âge de procréer par la promotion d'un mode de vie sain. Cette étude se déroule à Kisantu et ses environs. Vous avez été choisie pour participer à l'étude, et contribuer à sa réussite parce que vous habitez dans cette zone de santé, et que vous avez l'âge de procréation (18-49 ans). Votre participation à cette étude est très attendue car elle pourra permettre d'améliorer les connaissances sur le diabète chez la femme enceinte.

Grâce à cette étude, nous voulons savoir comment une dame ou un monsieur pourrait prévenir le diabète par une alimentation saine, activité physique et un poids idéal. Les résultats de cette étude peuvent sauver des vies en aidant à prévenir le diabète. Dans cette étude, il y aura environ 300 femmes de Kisantu et de ses environs, âgées de 18 à 49 ans qui seront divisées en deux groupes particuliers, un groupe aura diverses activités et l'autre groupe une seule activité au début de l'étude. Les activités comprendront des informations sur la prévention du diabète et du diabète gestationnel par un mode de vie sain. Les activités de votre groupe particulier comprennent une visite dans les ménages pour dispenser une éducation et une activité de groupe (par exemple, cours d'activité physique) tous les 6 moins. En outre, tous les 6 mois, vous serez invité à participer à des focus groups (groupes de discussion) pour explorer les perceptions de l'étude, identifier les besoins, les faiblesses et les domaines à améliorer. Ces focus groups resteront totalement anonymes. La participation à ces focus groups n'aura aucun impact sur les autres activités liées à l'intervention. Si vous acceptez de participer à l'étude, vous serez suivie par nos agents pour organiser les activités décrites ci-dessus. Le critère d'évaluation principal est un score de mode de vie sain mesuré par un questionnaire fermé. Les critères d'évaluation secondaire comprennent l'anthropométrie (poids, indice de masse corporelle et tour de taille), certains indicateurs cliniques (tension artérielle et glycémie) et d'autres paramètres sur l'alimentation et l'activité physique. Par ailleurs, vous aurez deux rendez-vous par an au centre de santé pour l'évaluation des paramètres. Le suivi durera deux ans. Vous n'aurez pas à payer pour les

Approved

procédures effectuées spécifiquement pour cette étude. Aucun remboursement ou compensation financière ne sera fourni. Nous souhaitons donc nous assurer que vous habitez dans la région, et que vous resterez toujours en contact avec le centre.
Cette étude présente un faible risque et vous bénéficierez de l'acquisition de connaissances concernant un mode de vie sain pour la prévention de la maladie chronique, d'un suivi plus étroit et de conseils individualisés.
Si vous êtes d'accord, veuillez signer ou apposer votre empreinte digitale en bas de la note de consentement. Nous vous remercions sincèrement pour votre participation à cette étude.

Partie 2. CONSENTEMENT DE LA PARTICIPANTE À L'ÉTUDE

Approved

(Français)
Je m'engage bénévolement de participer à cette étude sur la prévention du diabète sucré chez la femme en âge de procréer à Kisantu et aux activités demandées. J'ai eu des explications claires à ce sujet. J'ai reçu suffisamment d'informations orales et compréhensibles, avec suffisamment de temps pour poser des questions, et mes questions ont reçu une réponse adéquate.
Je sais que je peux arrêter ma participation à l'étude à tout moment sans que cela n'affecte le fait de continuer à utiliser le centre de santé et bénéficier de la même qualité de services et de soins de santé.
Je comprends que pendant ma participation à cette étude, mes données personnelles et médicales seront collectées et que la confidentialité de ces données est protégée conformément à la législation nationale, européenne et belge y afférente.
Je suis informé que mes données anonymisées ou pseudonymisées peuvent être transférées vers d'autres pays que la RD Congo (le cas échéant).
Je sais qu'en cas de nécessité, je peux appeler le numéro de téléphone suivant : 0998896442

Fait à ……………………………….., le ……./ …… /……….

Nom de la participante : ……………………………………………….   Nom du témoin : ……………………………

Signature (ou empreinte digitale) de la participante   Signature du témoin

Nom et signature du membre de l'équipe de l'étude : ……………………………………………………………………

(Kikongo)

Ndambu zole: NTAMBULULA YI NDONGOSOLO

Monontambuluele mu kala umosi ku bana bena mu ndongosolo yi kimbefo ki sukadi ku mwana n'kento yu una mu mbandu ku buta ku Kisantu ye mu bisalu bibalombele.

Mbakidi ntendula zi fwene. Mbakidi kinsunsa ki kudia biufu, ye mbakidi mvutu zina zi kieleka.

Nzeye nde ndendele yambula kio konso ntangu ye konda kiketa, ye ndendele fula mu kubaka lusalusu lu mavimpi ku lupitalu konda kiketa.

Ngindu zamo sa zikala mfunu mu tuadisa kio kisalu, mu diambu di nsi amo go mpi nsi zinkaka. Nzeye nde ngindu zamo zi lendi kwenda kwakulu,muna lutaninu luamo(kinsueki) ye konda kigonsa.

Go mfunu ngolo, ndendele mbokila mu nzila lami, mu nomba yi :………………………………………………………….

Masalamene ku……………………………………, le………/………/…………………………..

Zina ye ndiema nlembo yi mama | Zina ye ndiema nlembo yi mbangi

(Lingala)

Nandimi kokota na boyekoli ya bolukaluki mpo ya komibatela na bokono bwa sukali mpe nakofutama te. Boyekoli bona bokosalema epai ya basi ya Kisantu oyo bakokisa mibu ya kobota. Nazwaki mateya malongobani mpo na boyekoli bona. Nazwaki mpe tango ya kotuna mituna mpe bapesi nga biyano bisengeli.
Nayebi malamu ete nakoki kolongwa na boyekoli bona tango inso nalingi mpe kolongwa na ngai ekoki kozala na likama te mingi mingi na oyo etali kosalela lopitalo (Centre de Santé).
Nasosoli malamu ete na ntango ya boyekoli bona bakozwa mitango mpe makambo mosusu mpo na bokolongono bwa nzoto na ngai kasi makambo wana ekobombama sekele ndenge mibeko mya Kongo mpe mya mboka ya bapaya milobi.
Bayebisi nga ete mitango mpe makambo mosusu oyo ekobombama na ndenge ete mutu akoki kolimbola yanbo te ekoki kotindama na ba mboka mosusu soki esengeli.
Nayebi mpe ete soki nazali na motuna to na kokoso, nakoki kobenga na 0998896442 tango inso.

Esalemi na ……………………………. Mokolo mwa …./ ……./ …….

Kombo ya moyangani | Kombo ya motatoli

Mokoloto | Mokoloto

Kombo mpe mokoloto ya ba'oyo bazali kokamba boyekoli

Approved

Annex 5. *Informed consent form for participants in the comparison group*

Partie 1. FORMULAIRE D'INFORMATION PARTICIPANTE À L'ÉTUDE

Prévention des diabètes type 2 et gestationnel chez les femmes en âge de procréer à Kisantu, RD Congo

Vous êtes invitée à participer à une étude sur la prévention des diabètes type 2 et gestationnel chez la femme en âge de procréer, réalisée par MEMISA en collaboration avec l'Institut de Médecine Tropicale d'Anvers. Avant de décider de participer ou non à cette étude, il est important que vous compreniez les informations contenues dans ce formulaire. Ce formulaire explique vos droits, nos responsabilités, le but, les procédures, les avantages, risques et inconvénients éventuels, et votre droit de retirer votre consentement à tout moment.

N'hésitez pas à poser des questions, par exemple sur les avantages et risques éventuels concernant cette étude. Votre participation est entièrement volontaire. Vous pouvez parler de l'étude avec n'importe qui et prendre le temps de réfléchir à votre participation. Même si vous renoncez à participer à cette étude, vous continuerez à bénéficier des soins de qualité au sein de la formation sanitaire. Cependant, si vous choisissez d'y participer, vous devez toujours garder sur vous ces informations générales et le formulaire signé pendant toute la durée de l'étude.

OBJET ET DESCRIPTION DE L'ÉTUDE

Cette étude est menée pour en savoir plus sur la prévention du diabète chez la femme enceinte ou en âge de procréer par la promotion d'un mode de vie sain. Cette étude se déroule à Kisantu et ses environs. Vous avez été choisie pour participer à l'étude, et contribuer à sa réussite parce que vous habitez dans cette zone de santé, et que vous avez l'âge de procréation (18-49 ans). Votre participation à cette étude est très attendue car elle pourra permettre d'améliorer les connaissances sur le diabète chez la femme enceinte.

Grâce à cette étude, nous voulons savoir comment une dame ou un monsieur pourrait prévenir le diabète par une alimentation saine, activité physique et un poids idéal. Les résultats de cette étude peuvent sauver des vies en aidant à prévenir le diabète. Dans cette étude, il y aura environ 300 femmes de Kisantu et de ses environs, âgées de 18 à 49 ans qui seront divisées en deux groupes particuliers, un groupe aura diverses activités et l'autre groupe une seule activité au début de l'étude. Les activités comprendront des informations sur la prévention du diabète et du diabète gestationnel par un mode de vie sain. Dans votre groupe particulier, l'éducation et les informations seront fournies au début de l'étude uniquement. Le critère d'évaluation principal est un score de mode de vie sain mesuré par un questionnaire fermé. Les critères d'évaluation secondaire comprennent l'anthropométrie (poids, indice de masse corporelle et tour de taille), certains indicateurs cliniques (tension artérielle et glycémie) et d'autres paramètres sur l'alimentation et l'activité physique. Par ailleurs, vous aurez deux rendez-vous par an au centre de santé pour l'évaluation des paramètres. Le suivi durera deux ans. Vous n'aurez pas à payer pour les procédures effectuées spécifiquement pour cette étude. Aucun remboursement ou compensation financière ne sera fourni. Nous souhaitons donc nous assurer que vous habitez dans la région, et que vous resterez toujours en contact avec le centre.

Cette étude présente un faible risque et vous bénéficierez de l'acquisition de connaissances concernant un mode de vie sain pour la prévention de la maladie chronique, d'un suivi plus étroit et de conseils individualisés.

Approved

Si vous êtes d'accord, veuillez signer ou apposer votre empreinte digitale en bas de la note de consentement. Nous vous remercions sincèrement pour votre participation à cette étude.

Partie 2. CONSENTEMENT DE LA PARTICIPANTE À L'ÉTUDE

(Français)
Je m'engage bénévolement de participer à cette étude sur la prévention du diabète sucré chez la femme en âge de procréer à Kisantu et aux activités demandées. J'ai eu des explications claires à ce sujet. J'ai reçu suffisamment d'informations orales et compréhensibles, avec suffisamment de temps pour poser des questions, et mes questions ont reçu une réponse adéquate.
Je sais que je peux arrêter ma participation à l'étude à tout moment sans que cela n'affecte le fait de continuer à utiliser le centre de santé et bénéficier de la même qualité de services et de soins de santé.
Je comprends que pendant ma participation à cette étude, mes données personnelles et médicales seront collectées et que la confidentialité de ces données est protégée conformément à la législation nationale, européenne et belge y afférente.
Je suis informé que mes données anonymisées ou pseudonymisées peuvent être transférées vers d'autres pays que la RD Congo (le cas échéant).
Je sais qu'en cas de nécessité, je peux appeler le numéro de téléphone suivant : 0998896442

Fait à ………………………………….., le ……./ …… /……….

Nom de la participante : ……………………………………………….. Nom du témoin : ……………………………

Signature (ou empreinte digitale) de la participante Signature du témoin

Nom et signature du membre de l'équipe de l'étude : ……………………………………………………………………

Approved

(Kikongo)

Ndambu zole: NTAMBULULA YI NDONGOSOLO

Monontambuluele mu kala umosi ku bana bena mu ndongosolo yi kimbefo ki sukadi ku mwana n'kento yu una mu mbandu ku buta ku Kisantu ye mu bisalu bibalombele.

Mbakidi ntendula zi fwene. Mbakidi kinsunsa ki kudia biufu, ye mbakidi mvutu zina zi kieleka.

Nzeye nde ndendele yambula kio konso ntangu ye konda kiketa, ye ndendele fula mu kubaka lusalusu lu mavimpi ku lupitalu konda kiketa.

Ngindu zamo sa zikala mfunu mu tuadisa kio kisalu, mu diambu di nsi amo go mpi nsi zinkaka. Nzeye nde ngindu zamo zi lendi kwenda kwakulu,muna lutaninu luamo(kinsueki) ye konda kigonsa.

Go mfunu ngolo, ndendele mbokila mu nzila lami, mu nomba yi :…………………………………………………………

Masalamene ku……………………………………, le………/………/…………………………..

Zina ye ndiema nlembo yi mama **Zina ye ndiema nlembo yi mbangi**

(Lingala)

Nandimi kokota na boyekoli ya bolukaluki mpo ya komibatela na bokono bwa sukali mpe nakofutama te. Boyekoli bona bokosalema epai ya basi ya Kisantu oyo bakokisa mibu ya kobota. Nazwaki mateya malongobani mpo na boyekoli bona. Nazwaki mpe tango ya kotuna mituna mpe bapesi nga biyano bisengeli.
Nayebi malamu ete nakoki kolongwa na boyekoli bona tango inso nalingi mpe kolongwa na ngai ekoki kozala na likama te mingi mingi na oyo etali kosalela lopitalo (Centre de Santé).
Nasosoli malamu ete na ntango ya boyekoli bona bakozwa mitango mpe makambo mosusu mpo na bokolongono bwa nzoto na ngai kasi makambo wana ekobombama sekele ndenge mibeko mya Kongo mpe mya mboka ya bapaya milobi.
Bayebisi nga ete mitango mpe makambo mosusu oyo ekobombama na ndenge ete mutu akoki kolimbola yanbo te ekoki kotindama na ba mboka mosusu soki esengeli.
Nayebi mpe ete soki nazali na motuna to na kokoso, nakoki kobenga na 0998896442 tango inso.

Esalemi na ……………………………… Mokolo mwa …./ ……./ …….

Kombo ya moyangani Kombo ya motatoli

Mokoloto Mokoloto

Kombo mpe mokoloto ya ba'oyo bazali kokamba boyekoli

Annex 6. *Lifestyle questionnaire score weighted answers*

Question	Answers				
	A	B	C	D	E
Q1	1	1	2	2	2
Q2	1	2	3	4	5
Q3	1	2	3	4	5
Q4	1	2	3	4	5
Q5	1	2	3	4	5
Q6	1	2	3	4	5
Q7	5	4	3	2	1
Q8	5	4	3	2	1
Q9	1	2	3	4	5
Q10	1	2	3	4	5
Q11	1	2	3	4	5
Q12	1	1	2	2	3
Q13	5	4	3	2	1
Q14	5	4	3	2	1

Min score	14
Max score	65

Approved

Annex 7. WDD-W questionnaire

Required – Rows A–N (14 rows) will be aggregated during analysis into the ten MDD-W food groups			
	**Food categories**	**Description/examples to be adapted**<br>Consult **Appendix 2** and replace the example foods below with items commonly consumed in the survey area(s).	**Consumed**<br>Yes = 1<br>No= 0
**A**	**Foods made from grains**	*Porridge, bread, rice, pasta/noodles or other foods made from grains*	___yes (1)<br>___no (0)
**B**	**White roots and tubers and plantains**	*White potatoes, white yams, manioc/cassava/yucca, cocoyam, taro or any other foods made from white-fleshed roots or tubers, or plantains*	___yes (1)<br>___no (0)
**C**	**Pulses (beans, peas and lentils)**	*Mature beans or peas (fresh or dried seed), lentils or bean/pea products, including hummus, tofu and tempeh*	___yes (1)<br>___no (0)
**D**	**Nuts and seeds**	*Any tree nut, groundnut/peanut or certain seeds, or nut/seed "butters" or pastes*	___yes (1)<br>___no (0)
**E**	**Milk and milk products**	*Milk, cheese, yoghurt or other milk products but NOT including butter, ice cream, cream or sour cream*	___yes (1)<br>___no (0)
**F**	**Organ meat**	*Liver, kidney, heart or other organ meats or blood-based foods, including from wild game*	___yes (1)<br>___no (0)
**G**	**Meat and poultry**	*Beef, pork, lamb, goat, rabbit, wild game meat, chicken, duck or other bird*	___yes (1)<br>___no (0)
**H**	**Fish and seafood**	*Fresh or dried fish, shellfish or seafood*	___yes (1)<br>___no (0)
**I**	**Eggs**	*Eggs from poultry or any other bird*	___yes (1)<br>___no (0)
**J**	**Dark green leafy vegetables**	*List examples of any medium-to-dark green leafy vegetables, including wild/foraged leaves*	___yes (1)<br>___no (0)
**K**	**Vitamin A-rich vegetables, roots and tubers**	*Pumpkin, carrots, squash or sweet potatoes that are yellow or orange inside (see **Appendix 2** for other less-common vitamin A-rich vegetables)*	___yes (1)<br>___no (0)
**L**	**Vitamin A-rich fruits**	*Ripe mango, ripe papaya (see **Appendix 2** for other less-common vitamin A-rich fruits)*	___yes (1)<br>___no (0)
**M**	**Other vegetables**	*List examples of any other vegetables*	___yes (1)<br>___no (0)
**N**	**Other fruits**	*List examples of any other fruits*	___yes (1)<br>___no (0)

Approved

Annex 8. Focus Group Discussion (FGD) Guide

Planification and invitation

1. Organize a focus group meeting at each healthcare center, fix a suitable place for this activity. The estimated time is 1.5 hours.
2. Invite all enrolled participants in the program to their healthcare centers for a group discussion. Emphasize that participation is completely voluntarily and will not have an impact in other related-activities of the program.
3. Important to keep in mind that no names of participants attending to this activity will be written down.

Instructions for the focus group

1. Prior stating the focus group, divide the participants in groups of maximum of 10 participants.
2. Each group of a max. of 10 people should include at least one moderator, peer educator or healthcare professional. Ideally, two moderators per group, one to ask the questions and encourage discussion and 1 to take notes and write down the most salient discussion points.
3. The focus group should start with the moderator/s (peer educator and/or healthcare professional) introducing themselves and re-informing the participants that the main objective of focus group is to understand their perception regarding the program for the prevention of type 2 diabetes and gestational diabetes and also to identify needs, weaknesses and improvement points of the program. The conclusions made in this discussion will help us to address and improve the program and tailoring it towards your needs. Emphasize that the discussions is anonymous as no names of the attending participants will be written down and only overall points and conclusions will be written down.

Questions:

1. Do you think this program has been useful for you?
2. Have you put into practice any of the recommendations given by this program?
3. Until now, what have you liked the most regarding this program?
4. Until now, what have you not liked regarding this program?
5. In your opinion, what could be improved in this program?
6. In your opinion, what has not been useful in this program?
7. In your opinion, would you like to involve your families and other members of the community (e.g. government agencies, NGO's, community leaders, religious representatives) in this program?

Approved

Annex 9

Profile for peer educators:

- Female
- Live in the surrounding areas of the healthcare center
- Have achieved at least 4 years of secondary school
- Flexibility to mobilize
- Proven communication skills
- Request the advice of the Health Area Development Committees (CODESA) and Zones de Santé (BCZS).

Profile for health worker:

- Female, if possible
- Work as a health professional in one of the 6 healthcare centers of the study
- Collaborate among maternity, pre-natal consultations (CPN), child monitoring or outpatient consultation
- Agree to collaborate in the study

Approved
