# Supplementary material for: Improving lifestyle behaviours among women in Kisantu, the Democratic Republic of the Congo: A protocol of a cluster randomised controlled trial
Source: PLoS One. 2022 Sep 9;17(9):e0274517. doi: 10.1371/journal.pone.0274517 (PMC9462713; doi:10.1371/journal.pone.0274517)
Supplement: S3 File — (PDF) [file pone.0274517.s003.pdf]

S1 File. Healthy lifestyle questionnaire

Q1) How often do you eat meals in a day (including tea, coffee, fruits, vegetables, cassava, bread, taro, etc)?

A) >6 times
B) 6 times
C) 5 times
D) 4 times
E) 3 times

Q2) How often do you drink sweetened beverages like soft drinks, juices, etc.?

A) At least once daily
B) 3 to 6 times a week
C) 1 to 2 times a week
D) 2 to 3 times a month
E) Once a month or less

Q3) How often do you eat sweets such as cake, jam, chocolate, candy, cookies, etc.?

A) At least once daily
B) 3 to 6 times a week
C) 1 to 2 times a week
D) 2 to 3 times a month
E) Once a month or less

Q4) How often do you eat fried foods such as potato fries, sweet potato fries, fried cassava, fried plantains, fried chicken, etc?

A) At least once daily
B) 3 to 6 times a week
C) 1 to 2 times a week
D) 2 to 3 times a month
E) Once a month or less

Q5) How often do you eat high salty snacks such as salty peanuts, popcorn, chips, etc.?

A) At least once daily
B) 3 to 6 times a week
C) 1 to 2 times a week
D) 2 to 3 times a month
E) Once a month or less

Q6) How often do you consume sugar and honey in tea, coffee, etc.?

A) At least once daily
B) 3 to 6 times a week
C) 1 to 2 times a week
D) 2 to 3 times a month
E) Once a month or less

Q7) How often do you eat fruit and vegetables?

A) Every time in the main diet
B) At least once a day
C) 3 to 4 times a week
D) 1 time a week
E) Less than once a week.

Q8) How often do you eat sprouted pulses and green vegetables (cassava leaves, spinach, sorrel)?

A) Every time in the main diet
B) At least once a day
C) 3 to 4 times a week
D) 1 time a week
E) Less than once a week

Q9) How often do you eat saturated fat like mutton fat, egg yolks, butter, margarine, etc.?

A) At least once daily
B) 3 to 6 times a week
C) 1 to 2 times a week
D) 2 to 3 times a month
E) Once a month or less

Q10) How often do you eat refined food items like pastries, bread with cold meats + soda, etc.?

A) At least once daily
B) 3 to 6 times a week
C) 1 to 2 times a week
D) 2 to 3 times a month
E) Once a month or less

Q11) How often do you eat ghee, cream, mayonnaise, etc.?

A) At least once daily
B) 3 to 6 times a week
C) 1 to 2 times a week
D) 2 to 3 times a month
E) Once a month or less

Q12) How often do you eat out of the house (such as weddings, family reunions etc.)?

A) More than 3 times a week
B) More than once a week
C) 2 times in a month
D) 1 time in a month
E) Less than 1 time in a month

Q13) How many days do you exercise in a week?

A) Daily
B) 5 to 6 times a week
C) 3 to 4 times a week
D) 1 to 2 times a week
E) Never

Q14) How much time do you exercise for each session?

A) >40 minutes
B) 30–40 minutes
C) 20–30 minutes
D) 20–10 minutes
E) <10 minutes
