# Supplementary material for: Improving lifestyle behaviours among women in Kisantu, the Democratic Republic of the Congo: A protocol of a cluster randomised controlled trial
Source: PLoS One. 2022 Sep 9;17(9):e0274517. doi: 10.1371/journal.pone.0274517 (PMC9462713; doi:10.1371/journal.pone.0274517)
Supplement: Attachment — Submitted filename: Reponse to reviewers.docx [file pone.0274517.s004.docx]

**RESPONSES TO EDITORS AND REVIEWERS**

*We would like to thank the editors and reviewers for their comments and suggestions.*

*[responses are provided in italics]*

**Additional edit (requested 06/01/2022)**

1. Your ethics statement should only appear in the Methods section of your manuscript. If your ethics statement is written in any section besides the Methods, please move it to the Methods section and delete it from any other section. Please ensure that your ethics statement is included in your manuscript, as the ethics statement entered into the online submission form will not be published alongside your manuscript.

Please note that Ethics statement was also appear in the Abstract section of the manuscript.

***[Response]*** *We ha chosen to separate the previous ethics statement into two subsections of the Methods section 1) Ethics statement; 2) Confidentiality and risk (****Tracked manuscript: Methods section Ethics statement and Confidentiality and risks, lines 344-353, page 17; Clean version: Methods section Ethics statement and Confidentiality and risks, lines 340-348, page 17****). The current ethic statement included in the manuscript has been also modified in the online submission, and equals the ethic statement in the abstract (****Tracked manuscript: Abstract, lines 67-73, page 3-4; Clean version: Abstract, lines 63-69, page 3-4)****.*

**Additional edit (requested 06/01/2022)**

1. Your ethics statement should only appear in the Methods section of your manuscript. If your ethics statement is written in any section besides the Methods, please move it to the Methods section and delete it from any other section. Please ensure that your ethics statement is included in your manuscript, as the ethics statement entered into the online submission form will not be published alongside your manuscript.

***[Response]*** *The ethics statement has been moved to the Methods section (subheading Ethics statement). (****Tracked manuscript: Methods section Ethics statement, lines 344-345, page 17; Clean version: Methods section Ethics statement, lines 340-342, page 17****). Also, the ethics statement entered into the online submission has been modified to equal the ethics statement included in the manuscript.*

**Journal Requirements:**

1. Please ensure that your manuscript meets PLOS ONE's style requirements, including those for file naming. The PLOS ONE style templates can be found at 
<https://journals.plos.org/plosone/s/file?id=wjVg/PLOSOne_formatting_sample_main_body.pdf> and 
<https://journals.plos.org/plosone/s/file?id=ba62/PLOSOne_formatting_sample_title_authors_affiliations.pdf>

***[Response]*** *The manuscript and corresponding files have been modified according to PLOS ONE’s requirements.*

2. We note that the grant information you provided in the ‘Funding Information’ and ‘Financial Disclosure’ sections do not match.

When you resubmit, please ensure that you provide the correct grant numbers for the awards you received for your study in the ‘Funding Information’ section.

***[Response]*** *The manuscript contains the correct information regarding the funding. The funding information in the electronic portal has been updated accordingly.*

3. We note that you have indicated that data from this study are available upon request. PLOS only allows data to be available upon request if there are legal or ethical restrictions on sharing data publicly. For more information on unacceptable data access restrictions, please see <http://journals.plos.org/plosone/s/data-availability#loc-unacceptable-data-access-restrictions>.

In your revised cover letter, please address the following prompts:

a) If there are ethical or legal restrictions on sharing a de-identified data set, please explain them in detail (e.g., data contain potentially sensitive information, data are owned by a third-party organization, etc.) and who has imposed them (e.g., an ethics committee). Please also provide contact information for a data access committee, ethics committee, or other institutional body to which data requests may be sent.

***[Response]*** *Currently, datasets have not been yet generated or analysed as this manuscript describes the study protocol of ongoing research. After the study termination,* *data cannot be shared publicly because it entails sensitive information, however, data generated will be available upon request to the corresponding author and only after consultation with the involved ethical committees. Both The institutional Review Board of the Institute of Tropical Medicine (contact: irb@itg.be) and the Ethical Committee of the University of Kinshasa (contact:* [*espsec_unikin@yahoo.fr*](mailto:espsec_unikin@yahoo.fr)*), should review the request and approve any use of the data generated from this project outside the original scope of work, and identified research teams. The data derived from this study, even if pseudonymised, and deprived of person’s identifiers, contains sensitive information such as sex, age, marital status, profession, number of children, and personal lifestyle habits from which identification cannot be ruled out. This statement has been modified in the manuscript (****Tracked version: section Metadata, lines 30-38, page 2; Clean version: Section Methods/Design, lines 29-36, page 2****).*

b) If there are no restrictions, please upload the minimal anonymized data set necessary to replicate your study findings as either Supporting Information files or to a stable, public repository and provide us with the relevant URLs, DOIs, or accession numbers. For a list of acceptable repositories, please see <http://journals.plos.org/plosone/s/data-availability#loc-recommended-repositories>.

***[Response]*** *For this study, no data has been generated or analysed as this manuscript only describes the study protocol of ongoing research.*

**Reviewers comments**

**Reviewer #1**: This paper was a protocol of cRCT for improving lifestyle behavior in adult women of reproductive age. This protocol is a feasible and structured study, and has a relevant point in women.

Major comment

The aim of this study is to prevent T2D and GDM, and to develop and evaluate the multi-component health promotion program. The primary outcome of this study is ambiguous, because the primary outcome is usually hard endpoint. I recommend that the primary outcome is increasing weight or BMI, and incident of T2D or GDM, and that this secondary outcome is the adherence to healthy lifestyle, and clinical indicators. I think the objectives and outcomes in you study do not match.

***[Response]*** *We thank the reviewer for this comment. The scope of this study is, indeed, to instil healthy habits in women to ultimately prevent the onset of chronic conditions, particularly type 2 diabetes and gestational diabetes whose prevalence is on the rise in DRC. The study focuses on health promotion rather than disease prevention, approaching public health from an upstream perspective and hoping to counteract the early adoption of risk factors (suboptimal diet and physical activity levels) right at the start of their adoption. As such, to evaluate the intervention’s impact, our primary outcome should be a measure of our intervention target; increase adherence to a healthy lifestyle in women. We agree that measuring the adherence to a healthy lifestyle is less objective than a hard endpoint such as diagnosis of type 2 diabetes, however, we have chosen it to effectively evaluate the impact of our intervention. As a health promotion intervention, our target population is not limited to women with overweight or obesity; women with a normal weight can also be part of the study. Using the change in weight or BMI as a primary outcome would therefore imply that our intervention carries a major component of weight maintenance/reduction which is not the case, as our intervention components revolve around instilling healthy habits. Reinforcing the potentially greater effectiveness of health promotion interventions in our research context is also the lack of information on the prevalence of metabolic risk factors (increased BMI, hyperglycemia) in this population. By choosing a health promotion approach we will introduce positive concepts, focusing on keeping health rather than developing a disease, that increase the overall wellbeing of the population while allowing us to also gain the needed insight into the frequency of metabolic risk factors. Nevertheless, we are aware of the potential introduction of response bias in our primary outcome due to socially desirable bias but this bias should be greatly minimized by the introduction of the randomized control (****Tracked version: section Discussion, lines 381-383, page 18; Clean version: Section Methods/Design, line 376-378, page 18****).*

**Reviewer #2**: This is a protocol of a cluster randomised controlled trial to improving lifestyle behaviour among women in Kisantu, the Democratic Republic of the Congo. Well wittern, organized and very interesting. The protocol follows to the SPIRIT. I have only one comment to the authors.

1. What is the rationale to set the duration of intervention as 24 months for improving lifestyle behaviour? Changing lifestyle behaviour is usually challenging as the study participants are relcutant to accept new lifestyle behaviour and these lifestyle interventions usually reduce efficacy after finishing interventions (e.g. food intervention for hypetension etc...).

***[Response]*** *We thank the reviewer for the comment. We have set the duration of the programme to 24 months to guarantee we have the sufficient time to witness an improvement in lifestyle in the intervened clusters. This extended duration is uncommon in randomized trials, that tend to be of shorter duration and evaluate more intense interventions. In our case the cluster randomization allows us to maintain our control centers receiving usual care in a separate setting, offering the opportunity to implement a sustainable, context-based intervention for health promotion hoping for a gradual adoption of healthier habits. Throughout the 24 months and multiple follow up visits, we expect to see a gradual increase in healthy lifestyle measurement, in contrast with the controls that should remain constant. This gradual approach has been identified as the one with most potential to be adopted by the community and to be further uptake by health agencies in DRC, and as such as been backed up by the health district of Kisantu, DRC, and the national diabetes program (represented by Dr. Jean-Claude Dimbelolo in this work), as well as by the study sponsor and the Institute of Tropical Medicine’s IRB. (****Tracked version: section Methods/Design – Quality assurance, lines 284-285, page 14; Clean version: Section Methods/Design, line 280-281, page 14****). We expect this 24-month programme to be successful and thereafter for the health district of Kisantu investing in its continuation and integration into usual care. Additionally, we also aim at acquiring future funding for continuing this research, mainly related to evaluating the sustainability of the programme, scaling up the programme to target larger populations, and looking for additional avenues to maintain or increase the expected impact.*
