# Supplementary material for: Improving lifestyle behaviours among women in Kisantu, the Democratic Republic of the Congo: A protocol of a cluster randomised controlled trial
Source: PLoS One. 2022 Sep 9;17(9):e0274517. doi: 10.1371/journal.pone.0274517 (PMC9462713; doi:10.1371/journal.pone.0274517)
Supplement: Attachment — Submitted filename: Reponse to reviewers.docx [file pone.0274517.s005.docx]

**RESPONSES TO EDITORS AND REVIEWERS**

*We would like to thank the editors and reviewers for their comments and suggestions.*

*[responses are provided in italics]*

**Journal Requirements:**

Please review your reference list to ensure that it is complete and correct. If you have cited papers that have been retracted, please include the rationale for doing so in the manuscript text, or remove these references and replace them with relevant current references. Any changes to the reference list should be mentioned in the rebuttal letter that accompanies your revised manuscript. If you need to cite a retracted article, indicate the article’s retracted status in the References list and also include a citation and full reference for the retraction notice.

*[Response] We have reviewed the reference list and ensured it is complete and correct. Also we have ensured that none of the current cited references have been retracted. The following modifications were made, aside from formatting:*

- *Previous reference 9 was deleted as it was unrelated to the paper.*
- *Previous reference 18, currently 17, has been replaced with a relevant current reference.*
- *Previous references 25 and 28 were (previous submission) were deleted as they were duplicated references, just in another language.*

*We believe the current list is correct. Please, let me know if any other modification/update of the references is needed.*

**Reviewers comments**

**Reviewer #1**:

Authors returned to our comments, but you did not add to reasons or rationale in this manuscript.
Reviewer 2 has one comment to the authors “what is the rationale to set the duration of intervention as 24 months for improving lifestyle behavior?” I recommend that authors add the lines, “we have set the duration of the programme to 24 months to guarantee we have the sufficient time to witness an improvement in lifestyle in the intervened clusters.” in Evaluation design (p6).
I think the objectives and outcomes in your study do not match. I do not change my comment. I recommend that the primary outcome is increasing weight or BMI, and incident of T2D or GDM, and that this secondary outcome is the adherence to healthy lifestyle, and clinical indicators.
Authors described in the comments that “this study focuses on health promotion rather than disease prevention, ---- risk factors (increased BMI, hyperglycemia) in this population.” I recommend that authors state the reasons in outcome, in page 11.

*[Response] We thank the reviewer for these comment. As suggested, we have included the rationale for setting the duration of the programme to 24 months in the revised manuscript (****Tracked version: section Methods/Evaluation Design, lines 136-138, page 6; Clean version: Section Methods/Evaluation, line 138-140, page 7****).*

*We really appreciate the thoughts of the reviewer on in the paper because it has make us realize that our objectives and outcomes were not clear enough. Therefore we have clarified the objectives (****Tracked version: section Methods/Objectives, lines 118-123, page 6; Clean version: Section Methods/Objectives, line 120-125, page 6****) and the reasons for choosing adherence to a healthy lifestyle as the main outcome in the manuscript (****Tracked version: section Methods/Outcome, lines 211-217, page 11; Clean version: Section Methods/Outcome, line 212-218, page 12).*** *We hope we have solved any concerns regarding our objectives and primary outcome. Also important to mention that we are targeting an apparently healthy population, therefore we do not expect them to develop any of these metabolic conditions to a great extent in the allocated follow-up. Moreover, modifying the primary outcome is not feasible anymore as the study has been already registered in ClinicalTrials.gov (NCT05039307) and has begun in October 2021. The ongoing study includes a specific sample size calculation of 288 women to detect a significant change in the stated primary outcome with an appropriate statistical power. Nevertheless, reflecting based on the reviewers comments, we have realised the need of a longer study/longer follow-up or a second trial to observe the sustainability of the intervention and capture these long(er)-term hard outcomes (e.g. incidence of T2D) proposed by the reviewer, as they are of great relevance for public health strategies.*

**Reviewer #2**:

The authors addessed the comments appropreately and revised manuscript improved significantly. I do not have further comments to the authors.

*[Response] We thank the reviewer for this comment.*
