# Supplementary material for: Improving lifestyle behaviours among women in Kisantu, the Democratic Republic of the Congo: A protocol of a cluster randomised controlled trial
Source: PLoS One. 2022 Sep 9;17(9):e0274517. doi: 10.1371/journal.pone.0274517 (PMC9462713; doi:10.1371/journal.pone.0274517)
Supplement: Attachment — Submitted filename: Reponse to reviewers and editorial team.docx [file pone.0274517.s006.docx]

We thank the reviewers and editorial team for their comments and suggestions.

*[responses are provided in italics]*

**Additional comment editorial team (19/07/2022)**

Please note that funding information should not appear in the Acknowledgments section or other areas of your manuscript. We will only publish funding information present in the Funding Statement section of the online submission form. Please remove any funding-related text from the manuscript.

*[Response] The funding statement has been deleted from the Metadata in the revised manuscript* ***(Only visible in the Tracked manuscript: Section Metadata, lines 22-25, page 2; Clean Manuscript: Section Metadata, lines: 22 (not visible anymore), page 2****). In the Acknowledgments, we only thank the sponsor of this research, The City of Antwerp, for their financial support.*

**Reviewer #1**:

I do not have further comments to the authors. The authors addressed back in response to our comments. This paper is better improved.

*[Response] We thank the reviewer for the comment.*

**Reviewer #3:**

In this study protocol, a two-arm cluster randomized-controlled trial is being proposed to educate women about healthy lifestyles and to ultimately prevent the onset of type 2 diabetes and gestational diabetes. The primary outcome is adherence to a healthy lifestyle as measured by a questionnaire. Secondary outcomes will include anthropometric measures and level of physical activity.

*[Response] We appreciate the reviewer’s comment.*

**Minor revisions:**

1- Abstract: Clarify the statement: “Data will be summarized and quantity using statistical mixed models.” What statistical methods will be used to summarize the data? What outcomes will be modeled using mixed linear regression analyses?

*[Response] Thanks for pointing this out. We have clarified how the data will be summarised and which outcomes will be quantified by the mixed models in the revised manuscript (****Tracked version: section Abstract, lines 62-64, page 3; Clean version: Section Abstract, line 62-65, page 3).***

2- Line 337: Typographical error: drop-outs

*[Response] The typographical error has been corrected in the revised manuscript (****Tracked version: section Methods/Power calculation, line 334, page 16; Clean version: Section Methods/Power calculation, line 338, page 17).***

3- Line 338: Remove the word ‘p-value’ from this line. Simply state “significance (one-sided), ...”

*[Response] The word ‘p-value’ has been removed in the revised manuscript (****Tracked version: section Methods/Power calculation, line 335, page 16; Clean version: Section Methods/Power calculation, line 339, page 17).***

4- Line 345: If the data is not normally distributed, differences in the central tendencies rather than means is typically investigated. If the data is categorical, chi-square or Fisher’s exact tests are typically used to compare differences between groups.

*[Response] Thank you for pointing this out. Indeed, we will use chi-square for categorical data. We have addressed the comment in the revised manuscript (****Tracked version: section Methods/Data analysis, line 343, page 16; Clean version: Section Methods/Data analysis, line 347, page 17).***

5- Line 351: Indicate the type of covariance structure that will be used in the mixed model and/or the criteria for selecting it.

[Response] We have clarified the type of covariance structure we will use for the mixed model in the revised manuscript *(****Tracked version: section Methods/Data analysis, line 351, page 16; Clean version: Section Methods/Data analysis, line 355, page 17).***

6- Ling 358: Drop “probability of” from this line.

*[Response] The word ‘probability’ has been removed in the revised manuscript (****Tracked version: section Methods/Data analysis, line 356, page 16; Clean version: Section Methods/Data analysis, line 360, page 18).***

7- Line 358: Consider replacing the sentence beginning with, “If multiple comparisons” with “Multiple comparisons will be adjusted by the Bonferroni method.”

*[Response] The suggested sentence has been added to the revised manuscript (****Tracked version: section Methods/Data analysis, lines 356-357, page 16; Clean version: Section Methods/Data analysis, lines 360-361, page 18).***

8- Indicate what software is being used to capture the data.

[Response] The software being used to capture the data has been added to the revised manuscript *(****Tracked version: section Methods/Data analysis, lines 358-359, pages 16-17; Clean version: Section Methods/Data analysis, lines 361-363, page 18).***
