# Supplementary material for: Improving lifestyle behaviours among women in Kisantu, the Democratic Republic of the Congo: A protocol of a cluster randomised controlled trial
Source: PLoS One. 2022 Sep 9;17(9):e0274517. doi: 10.1371/journal.pone.0274517 (PMC9462713; doi:10.1371/journal.pone.0274517)
Supplement: Attachment — Submitted filename: Reponse to reviewers and editorial team.docx [file pone.0274517.s007.docx]

We thank the reviewer and editorial team for their comments and suggestions.

*[responses are provided in italics]*

**Reviewer #3:**

Minor revisions: (Line numbers refer to those in the tracked changes version of revision 3.)

1. Line 64: Improve the grammar in the following sentence to make it more understandable. The term "quantity" causes confusion. "The primary and secondary outcomes will be quantity using statistical mixed models."

*[Response] The grammar of the sentence has been modified and clarified in the revised manuscript* ***(Tracked version: section Abstract, lines 63-64, page 3; Clean version: Section Abstract, line 64-65, page 3).***

2. Line 342: The following is a run-on sentence. "Demographic data will be explored for differences at baseline by comparing and testing means, if categorical data chi-square statistic will be used, of all demographic and baseline data between the groups." Consider the following revision. Group differences in baseline demographics will be compared with t-tests or chi-square tests.

*[Response] The suggested sentence has been incorporated to the revised manuscript* ***(Tracked version: section Abstract, lines 341-344, page 16; Clean version: Section Abstract, line 345-346, page 17).***

3. Thoroughly proofread the entire manuscript.

*[Response] The entire manuscript has been proofread, small grammar modifications have been made throughout the manuscript.*
